# Soil substrate culturing approaches recover diverse members of *Actinomycetota* from desert soils of Herring Island, East Antarctica

**DOI:** 10.1007/s00792-022-01271-2

**Published:** 2022-07-13

**Authors:** Nicole Benaud, Devan S. Chelliah, Sin Yin Wong, Belinda C. Ferrari

**Affiliations:** School of Biotechnology and Biomolecular Sciences, UNSW Sydney, Sydney, 2052 Australia

**Keywords:** Culturing, Natural products, Antimicrobial, Psychrotroph, Cold-adapted bacteria, Antarctic, *Streptomyces*

## Abstract

Antimicrobial resistance is an escalating health crisis requiring urgent action. Most antimicrobials are natural products (NPs) sourced from *Actinomycetota*, particularly the *Streptomyces*. Underexplored and extreme environments are predicted to harbour novel microorganisms with the capacity to synthesise unique metabolites. Herring Island is a barren and rocky cold desert in East Antarctica, remote from anthropogenic impact. We aimed to recover rare and cold-adapted NP-producing bacteria, by employing two culturing methods which mimic the natural environment: direct soil culturing and the soil substrate membrane system. First, we analysed 16S rRNA gene amplicon sequencing data from 18 Herring Island soils and selected the soil sample with the highest *Actinomycetota* relative abundance (78%) for culturing experiments. We isolated 166 strains across three phyla, including novel and rare strains, with 94% of strains belonging to the *Actinomycetota*. These strains encompassed thirty-five ‘species’ groups, 18 of which were composed of *Streptomyces* strains. We screened representative strains for genes which encode polyketide synthases and non-ribosomal peptide synthetases, indicating that 69% have the capacity to synthesise polyketide and non-ribosomal peptide NPs. Fourteen *Streptomyces* strains displayed antimicrobial activity against selected bacterial and yeast pathogens using an in situ assay. Our results confirm that the cold-adapted bacteria of the harsh East Antarctic deserts are worthy targets in the search for bioactive compounds.

**Supplementary Information:**

The online version contains supplementary material available at 10.1007/s00792-022-01271-2.

## Introduction

Culture-dependent approaches are known to vastly underestimate soil microbial diversity (Amann et al. 1995; Cary et al. 2010; Ferrari et al. 2008; Lewis 2013). Nevertheless, microbial isolation remains critical to downstream analysis across numerous scientific fields including natural product discovery. Low molecular weight organic molecules, or natural products (NPs), have been the source of one third of all small molecule drugs approved in medicine over the last 40 years (Newman and Cragg 2020; Xiangyang et al. 2010). These include therapeutically important drugs such as antimicrobials (e.g. chloramphenicol, daptomycin), cancer therapeutics (e.g. daunomycin, bleomycin) and immunosuppressive agents (e.g. rapamycin, cyclosporine), which have predominantly been derived from bacteria and fungi, and synthesised via polyketide synthase (PKS) and non-ribosomal peptide synthetase (NRPS) pathways (Cragg and Newman 2013; Harvey et al. 2015). Historically, filamentous *Actinomycetota*, most notably the *Streptomyces* genus, have provided the richest source of bacterial NPs (Bérdy 2005; Baltz 2007; Oren and Garrity 2021). Other prolific NP-synthesising bacteria include the *Myxococcota* such as the predatory *Myxococcales*; the *Pseudomonadota* genus *Pseudomonas*; the *Bacillota* genus *Bacillus*; and *Cyanobacteria* such as the *Nostoc* and *Anabaena* genera (Masschelein et al. 2017; Bérdy 2005; Burja et al. 2001). More recently, rarely cultured and candidate phyla such as the *Planctomycetota*, *Chloroflexota*, *Acidobacteriota*, *Verrucomicrobiota*, *Ca*. *Dormibacterota* and *Armatimonadota* have emerged as worthy targets for novel NP discovery, owing to increasingly available genomic sequencing data which has revealed the presence of biosynthetic gene clusters (BGCs) in these underexplored taxa (Crits-Christoph et al. 2018; Sharrar et al. 2019; Wiegand et al. 2020; Ji et al. 2017).

In the search for new therapeutics, there is an increasing focus on metabolites from rare or novel species residing in extreme habitats, such as hot and cold deserts, caves, oceans and hypersaline lakes (Bull and Goodfellow 2019; Millán-Aguiñaga et al. 2019). Extreme environments pose extraordinary survival challenges to life and consequently harbour metabolically unique microorganisms, in addition to remaining underinvestigated (Rateb et al. 2018; Bowman 2018). Extremophile bioprospecting has led to the discovery of at least 400 new chemical structures (Sayed et al. 2020): for example, atacamycins and the salternamides, polyketide groups which were derived from *Streptomyces* spp. isolated from the Atacama desert and a hypersaline pool, respectively (Kim et al. 2015; Nachtigall et al. 2011). Polar and subpolar environments offer enormous potential for NP discovery given the myriad of environmental stresses to which resident microbiota are exposed and the high proportion of *Actinomycetota* and novel taxa within Antarctic cold deserts (Ji et al. 2022). At least 13 novel NPs have been reported from Antarctic bacteria thus far, for example, the antitumour antibiotic frigocyclinone (Tian et al. 2017; Bruntner et al. 2005). More recently, targeted recovery of spore-forming *Actinomycetota* from Antarctic and sub-Artic sediments using MS/MS analyses revealed new specialized metabolites within rare *actinomycetes*, suggesting that polar bacteria harbour considerable novel chemical potential (Millán-Aguiñaga et al. 2019).

Traditional culturing methods which rely on serial liquid dilutions and plating to nutrient-rich artificial media rarely select for novel oligotrophic soil bacteria, even within the well characterised *Actinomycetota* and *Pseudomonadota* phyla (Jensen and Mafnas 2006; Nichols et al. 2010; Janssen et al. 2002; Zengler 2009). Here, we employed two culturing methods which mimic the natural environment: direct soil culturing (DSC) (Shimkets et al. 2006), and the soil substrate membrane system (SSMS) (Ferrari et al. 2008), with the aim of selecting for rare and cold-adapted NP-producing bacteria from Antarctic soil, specifically targeting *Actinomycetota* and *Myxococcota*. DSC was adapted from *Myxococcota* cultivation methods which exploit the ability of the taxa to form fruiting bodies. These fruiting bodies are visualised by stereomicroscopy atop natural substrates such as soil and wood particles, and give rise to predatory cells which swarm towards bait sources such as *Escherichia coli* or cellulose (Shimkets et al. 2006; Karwowski et al. 1996; Dawid et al. 1988; Gaspari et al. 2005). While *Myxococcota* are ubiquitous in soil, descriptions of cold-adapted members remain rare (Wenzel and Müller 2009; Herrmann et al. 2017; Dawid et al. 1988). Interestingly, the production of antimicrobial metabolites is thought to play a role in epibiotic predation, and predatory behaviour has also been observed in *Streptomyces* species (Kumbhar et al. 2014). The second method of cultivation was the soil substrate membrane system (SSMS), a culturing approach developed for soil which has enabled recovery of new species of *Pseudomonadota*, *Actinomycetota* and *Bacteroidota* (van Dorst et al. 2016; Ferrari et al. 2005). Additionally, the SSMS has been found to enrich difficult to isolate classes such as *Saccharimonadia* (formerly TM7), and *Chlorobia*, as well as rare phyla known to harbour BGCs, including *Gemmatimonadota*, *Chloroflexota*, and *Verrucomicrobiota* (Ferrari et al. 2005; van Dorst et al. 2016; Wang et al. 2014). Here, the SSMS was modified for incubation under psychrophilic temperatures to recover cold-adapted strains.

**Fig. 1:**
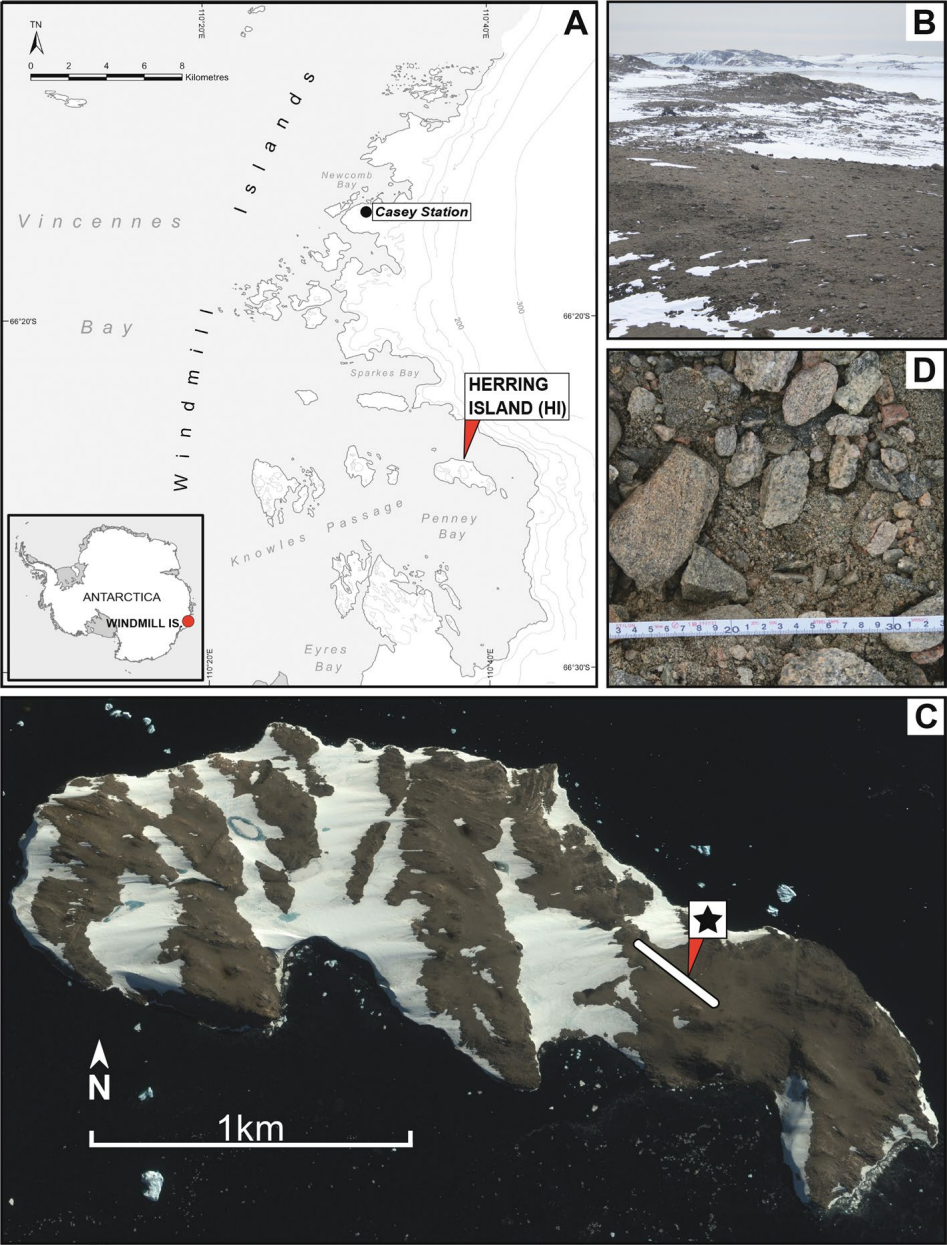
Herring Island, a barren Antarctic desert, located in the Windmill Islands region of East Antarctica. **A** Map of Windmill Islands, East Antarctica, showing the location of Herring Island in relation to Casey station. (Inset) The Antarctic continent, indicating the location of the Windmill Islands. **B** Photograph of the Herring Island sampling site. **C** Satellite image of Herring Island showing the location of the sampling region. Soils were sampled along three 300 m-long parallel transects at the location indicated by a line. The soil selected for culturing in this study was collected from the 200 m distance point, shown by a star. **D** Close-up photograph of sampled Herring Island soil used for culturing. Photographs supplied by the Environmental Protection Program, Australian Antarctic Division

Previously, we surveyed polar desert soils for bacterial PKS and NRPS encoding genes, and our work indicated that the pristine, low fertility desert soils from the Windmill Islands and Vestfold Hills regions of East Antarctica had novel NP biomining value (Benaud et al. 2019). Here, we selected soil from one of those locations for culturing experiments. Based on our previous NP gene amplicon sequencing study, this soil community contained a diversity of biosynthetic domain sequences, the majority of which displayed < 70% identity to known sequences (Benaud et al. 2019). Herring Island (HI) is an ice-free island situated approximately 15 km south of Casey station in the Windmill Islands, East Antarctica. The barren landscape of HI is composed primarily of garnet-bearing granite gneiss rock formed approximately 1250 million years ago, with the landscape displaying evidence of geologically recent deglaciation (< 8000 years ago) (Goodwin 1993; Bailey et al. 2016; Paul et al. 1995). HI is frequented by petrel seabirds, but is remote from human activity (Paul et al. 1995; Bailey et al. 2016, AADC 2018) (Fig. 1A). At HI, vascular plant life is absent (Fig. 1B). This barrenness is driven by sub-zero temperatures, high aridity and low humidity, resulting in limited concentrations of soil carbon, nitrogen and phosphorous, and reduced microbial biodiversity in comparison to temperate soils (Siciliano et al. 2014; Cary et al. 2010; Maestre et al. 2015). HI soils have not been the focus of previous bacterial culturing reports.

## Materials and methods

### Herring Island soil collection and 16S rRNA gene amplicon sequencing analysis

Soils from the HI site were sampled in 2006 as part of a larger biodiversity project, with the soils collected along a geospatial sampling design comprising three parallel 300 m-long transects (Fig. 1C) (van Dorst et al. 2014; Siciliano et al. 2014). Soils were stored at −80 °C until analysis.

DNA extraction, bacterial 16S rRNA gene fragment amplification and sequencing analysis for the 18 HI soils were performed as previously described (van Dorst et al. 2014; Siciliano et al. 2014). Briefly, DNA was extracted using the FastDNA™ SPIN Kit for Soil (MP Biomedicals, Seven Hills, Australia), and amplicon sequencing performed using the primer set 27F and 519R. Sequencing was performed using the 454 FLX titanium platform. Operational taxonomic units (OTUs) were clustered using ≥ 97% similarity (Siciliano et al. 2014; van Dorst et al. 2014; Ferrari et al. 2015).

For the current study, OTUs were taxonomically classified against the SILVA v138 SSU rRNA database (Quast et al. 2012), and relative abundance of bacterial 16S rRNA gene sequences for each of 18 HI soils calculated, first at phyla level, then for *Actinomycetota* at genus level, and visualised as stacked bar charts in R 3.4.0 using the ggplot2 package 2.2.1 (Wickham 2009). The determination of differential abundant bacterial phyla between HI transect communities was performed using *analysis of composition of microbiomes with bias correction* (ANCOM-BC) (Lin and Peddada 2020). ANCOM-BC estimates a change between test groups for each taxon using log-transformed values of absolute sequence counts. All phyla were included in the analysis, with data from each transect pooled by distance (e.g. ANCOM-BC_100m: Transects 1, 2 and 3 at 100 m distance) (SI Table 2). Results were corrected for multiple comparisons using the Holm–Bonferroni method, controlling the false discovery rate (fdr).

### Direct soil culturing methods (DSC)

#### Soil and culture plate preparation

Soil from the second transect (T2) at the 200 m distance point (HI/T2/200) was selected for culturing (Fig. 1D) (https://doi.org/10.4225/15/526F42ADA05B1). This soil was low in carbon content (600 ppm) and moisture (3.2%) and had a near-neutral pH of 6.6 (SI Table 1) (van Dorst et al. 2014; Siciliano et al. 2014). One portion of the HI/T2/200 soil (1.5 g) was divided into two treatments, designated ‘pre-treated’ and ‘untreated’ (SI Fig. 1). The pre-treated soil (0.5 g) was defrosted at RT (~ 21 °C), air dried in covered Petri plates at 37 °C for 30 min, then suspended in 3.5 mL sterile Milli-Q^®^ water (Merck Millipore, Burlington, MA USA). The soil–water suspension was sonicated (XUBA1, Grant, Cambridge, UK) at 44 kHz for 1 min, then incubated in a water bath at 56 °C for 10 min (Karwowski et al. 1996). This pre-treatment was hypothesised to select for mild heat- and sonication-resistant spore-forming microorganisms such as *Streptomyces* and *Myxococcota* (Karwowski et al. 1996; Daza et al. 1989). For the untreated soil, 1 g of soil was defrosted at 4 °C, suspended in 500 µL of sterile Milli-Q water and briefly vortexed before use. Water agar plates (WCX) (per litre: 1 g CaCl_2_·2H_2_O, 15 g agar (Sigma-Aldrich, St. Louis, MO, USA) were prepared with cycloheximide (R&D Systems, Minneapolis, MN, USA) added to suppress fungal growth at concentrations of 50 µg/mL (untreated soil) and 25 µg/mL (pre-treated soil) (Shimkets et al. 2006).

#### DSC baiting methods

For the *Escherichia coli* lawn baiting method, *E. coli* ATCC 25922 was cultured overnight at 37 °C on nutrient agar (NA) (per litre: 13 g nutrient broth, 15 g agar) (Oxoid, Thermo Scientific, Waltham, MA, USA). A large loopful of *E. coli* was transferred into 1 mL 0.9% saline to create a dense suspension which was applied as three circular lawns to two WCX agar plates (SI Fig. 1) and allowed to dry at RT. For the cellulose baiting method, sterile 10 mm diameter Whatman^®^ grade 1 filter paper discs (Whatman, Buckinghamshire, UK) were applied in groups of three to two WCX agar plates (SI Fig. 1) (Dawid et al. 1988; Shimkets et al. 2006). Portions of pre-treated or untreated soil (~ 10 mm diameter) were then applied onto the bait sources using a sterile spatula (SI Fig. 1). Plates were wrapped in parafilm and incubated at RT in the dark, for up to eight months, with small amounts of sterile water added periodically to maintain moisture.

#### Incubation and sub-culturing of DSC colonies onto secondary media

All plates were examined every 1–3 days throughout the incubation period under a stereomicroscope (40 × magnification) (Olympus, SZ40, North Ryde, Australia) and light microscope (100 × magnification) (Olympus, CH2, North Ryde, Australia), for fruiting body or colony formation. Colonies that had grown to sufficient size to be visible under microscopy were picked directly using a sterile toothpick and sub-cultured onto fresh WCX plates with *E. coli* or cellulose bait (Shimkets et al. 2006), and two additional media: 0.75 × NA (Oxoid) and soil extract with gellan gum (SEG) (per litre: 500 mL soil extract (per litre: 500 g Antarctic bulk soil Casey station test plot 123,567) and 7.2 g gellan gum (Gelzan™ Gelrite^®^, Sigma-Aldrich) with 1 g CaCl_2_·2H_2_O added to provide divalent cations required for gelling (James 1958), and incubated at RT in the dark. All cultured isolates were initially screened via Gram staining to determine cell morphology and culture purity and to eliminate fungal strains from further analysis (Beveridge 2001).

### SSMS culturing at cold temperatures

**Fig. 2:**
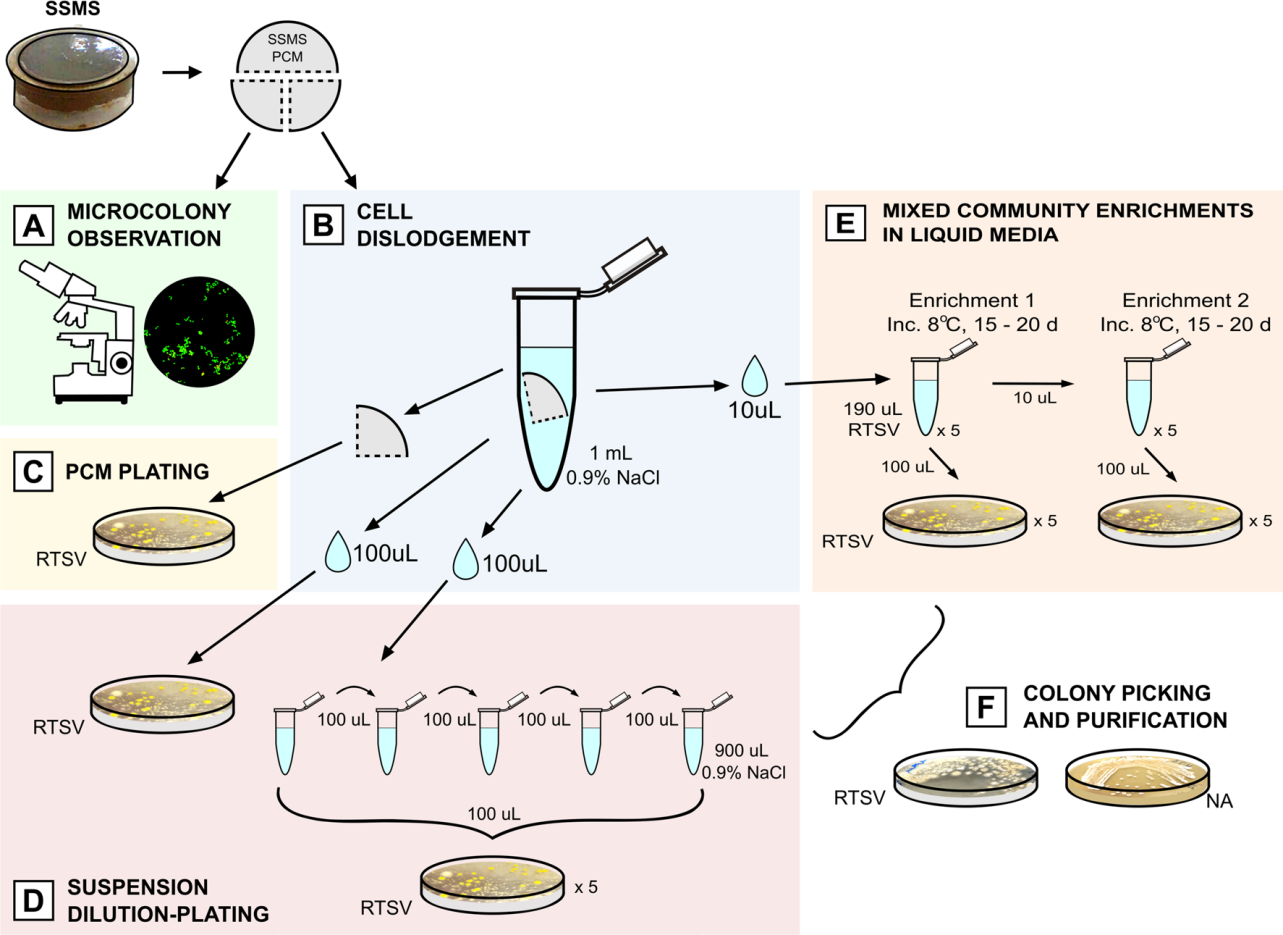
Flowchart depicting cold-incubated SSMS bacterial cultivation methods. **A** Portion of the polycarbonate membrane (PCM) was removed and microcolony growth and viability assessed by epifluorescence microscopy using a live/dead stain. **B** Portion of the PCM was vortexed with 0.9% NaCl to dislodge and suspend cells. **C** PCM was removed from NaCl and rubbed over the surface of 0.05 × RAVAN media with trace salts, vitamins and gellan gum (RTSV). **D** The cell suspension was serially diluted and spread-plated. **E** The cell suspension was passed into two rounds of mixed community enrichment in RTSV broth and spread-plated. **F** Resulting colonies were sub-cultured to RTSV media for pure isolation, then 0.75 × nutrient media (NA) for maintenance

Another portion of the HI/T2/200 soil (16.5 g) was used for culturing via the SSMS, adapted from Ferrari et al. (2008) with some additional modifications for psychrophilic conditions (Fig. 2). All equipment and reagents were equilibrated to 4 °C prior to use, and 4–8 °C temperatures were maintained throughout the entire experiment. The soil was defrosted at 4 °C. Tissue culture inserts (TCI) (Millicell^®^, 30 mm, polycarbonate, 0.4 µm, Millipore, North Ryde, Australia) were filled with a homogenous soil slurry prepared by vortexing 4.5 g HI soil with ~ 300 µL of 0.9% NaCl (Ajax Finechem, Taren Point, Australia) until the slurry evenly covered the filter membrane. Due to the low carbon content and rocky soil, the slurry was then secured against the membrane by filling the remaining TCI space with gellan gum (5 g/ L), which additionally facilitated moisture retention throughout the incubation period. SSMS cultures were prepared in triplicate, with inverted TCIs placed filter side up into a 6-well culture plate and incubated at 4 °C while the inoculum was prepared.

For the inoculum, 3 g of the HI/T2/200 soil was added to 27 mL 0.9% NaCl and vigorously vortexed for 10 s. Large particles were allowed to sediment at 4 °C for 1 min. A 1:100 dilution was then prepared by adding 100–900 µL 0.9% NaCl. For each triplicate culture, a 25 mm diameter, 0.22 µm pore size, hydrophilic polycarbonate membrane (PCM) (Isopore^®^, Millipore) was placed onto a moistened 25 mm diameter glass fibre filter (Whatman) on a sample filtration manifold (Carbon 14 Centralen, Hørsholm, Denmark) fitted with Millivac-Mini vacuum pump (Millipore). A 20 mL sterile stainless-steel cylinder was then secured and filled with 10 mL 0.9% NaCl and 50 µL of 1:100 inoculum and filtered onto PCM replicates. Each PCM was then applied to an inverted TCI membrane, ensuring complete contact, and the TCIs inserted into the 6-well plate. Sterile water was added to the plates’ outer wells to maintain hydration of cultures (Ferrari et al. 2008). The plate was sealed with Parafilm and incubated at 4 °C, for a total of 162 days.

#### Assessing microcolony growth and bacterial viability on the SSMS

Microcolony growth and viability was assessed at 50, 78 and 162 days of incubation (Fig. 2A). To confirm growth of microcolonies, ¼ PCM from one TCI replicate was abstracted using a sterile razor blade and secured to a microscope slide with 0.1% agarose (Bioline, Narellan, Australia). The PCM portion was treated with 1 drop (~ 25µL) of Vectashield mounting medium (Vector Laboratories, Burlingame, CA, USA), and a 1:1 ratio of Ultrapure™ water and the LIVE/DEAD^®^ BacLight™ Bacterial Viability stain (Invitrogen, Carlsbad, CA, USA) (Ferrari et al. 2005), and incubated at 4 °C in the dark for 30 min. PCM portions were then observed via epi-fluorescent microscopy using an Olympus BX51 microscope with DP74 camera (Olympus, North Ryde, Australia) and filters appropriate for excitation/emission maxima of 480/500 nm for SYTO 9 and 490/635 nm for propidium iodide (PI). When stained with the SYTO 9 and PI nucleic acid stains, live intact cells fluoresce green, while damaged and dead cells fluoresce red.

#### Secondary cultivation of SSMS microcolonies using artificial media

RAVAN media with trace salts and vitamins (RTSV) was used for secondary cultivation, comprising 0.05 × RAVAN (Watve 2000) with gellan gum as a solidifying agent (per litre: 1 g MgCl_2_·7H_2_0 and 7.2 g Gelzan (Sigma-Aldrich). Supplemental trace salts (per litre: 1 mg FeSO_4_·7H_2_O, 1 mg MnCl_2_·4H_2_O and 1 mg ZnSO_4_·7H_2_O) and Wolfe’s vitamin solution (per litre: 5 µg pyridoxine hydrochloride, 2.5 µg thiamine–HCl, 2.5 µg riboflavin, 2.5 µg nicotinic acid, 2.5 µg calcium d-( +)-pantothenate, 2.5 µg p-aminobenzoic acid, 2.5 µg thioctic acid, 1 µg biotin, 1 µg folic acid and 0.05 µg vitamin B12) were added after cooling. RAVAN is a low-concentration culturing medium designed to select for oligophilic bacteria (Watve 2000), and it was modified here with the aim to optimise recovery of *Actinomycetota* as well as novel species, and to promote sporulation in *Streptomyces* spp. (Hayakawa and Nonomura 1987; Zotchev et al. 2008; Wolin et al. 1963; Shirling and Gottlieb 1966). Gellan gum has been shown to benefit capture of rarely cultured environmental bacteria, including phyla such as *Gemmatimonadota* (Tanaka et al. 2014; Janssen et al. 2002).

To dislodge cells from the SSMS, PCM portions were placed in sterile 1.5 mL tubes containing 1 mL 0.9% NaCl and vortexed for 1 min (Fig. 2B). After removal from the cell suspension, PCMs were then plated by direct application over the surface of an 8 °C-equilibrated RTSV plate (Fig. 2C), wrapped in Parafilm and incubated at 8 °C. Cell suspensions were serially diluted by transferring 100 µL cell suspension to 900 µL 0.9% NaCl (Fig. 2D). Cell suspensions and serial dilutions (100 µL) were then spread plated onto 8 °C-equilibrated RTSV plates, wrapped in Parafilm and incubated at 8 °C. Cell suspensions were additionally used for liquid media enrichments, with 10 µL aliquots transferred to 0.2 mL tubes containing 190 µL RTSV broth (Fig. 2E). Enrichments were incubated at 8 °C for 15–20 days, then 100 µL aliquots was spread-plated onto 8 °C-equilibrated RTSV plates, wrapped in parafilm and incubated at 8 °C. A further 10 µL of the enrichment was used to inoculate fresh RTSV broth for a second enrichment round, with the incubation and plating procedure repeated.

#### Isolation and purification of bacteria from SSMS cultures

Spread-plated cultures (Fig. 2C–E) were regularly observed for growth, with incubation ranging between 27 and 347 days at 8 °C. Visible colonies were picked using a 1 µl sterile loop and sub-cultured onto solid RTSV until pure colonies were obtained (Fig. 2F). Once established in pure culture, isolates were tested for the ability to grow on high nutrient media (0.75 × NA) at 8 °C, followed by RTSV and 0.75 × NA plates at RT. Gram staining was performed on all SSMS-cultured isolates (Beveridge 2001).

### Bacterial isolate DNA extraction and purification

Genomic DNA was extracted from pure isolate subcultures grown on NA or RTSV plates using a bead-beating approach, followed by ethanol precipitation. A single large bacterial colony was transferred to a 2 mL screw-top microcentrifuge tube (Sarstedt AG and Co., Nümbrecht, Germany), containing 1 mL autoclaved Milli-Q water, and 0.5 g of an equal proportion 0.1 mm and 0.5 mm diameter glass beads (Mo Bio, Carlsbad, CA, USA). The mixture was homogenised using the FastPrep^®^-120 homogenisation instrument (MP Biomedicals, Irvine, CA, USA) for 40 s, on speed setting 6.0, and incubated for 5 min at 95 °C. Samples were centrifuged at 20,800×*g* for 3 min, and the supernatant was collected.

For ethanol precipitation, 1/10 volume of 3 M sodium acetate (CH_3_COONa, pH 5.2) was added to the DNA lysates, followed by two volumes of ice-cold 100% EtOH. DNA was precipitated at 8 °C for 20 min, then centrifuged at 17, 900×*g* for 20 min, and the supernatants were discarded. Pellets were re-suspended in 1 mL fresh 70% EtOH, and centrifuged at 17,900×*g* for 5 min. Following removal of supernatants, pellets were dried on a heat block for 15 min at 55 °C, and re-suspended in 150 μL TE buffer (10 mM Tris–HCl (pH 8.0), 0.1 mM EDTA). Genomic DNA was quantified using Nanodrop and stored at − 20 °C until further use.

### Taxonomic classification of pure strains via PCR amplification and Sanger sequencing of 16S rRNA genes

Taxonomic identification of strains was performed based on Sanger sequencing of the 16S rRNA gene. Near full-length (~ 1400 bp) 16S bacterial rRNA genes were PCR amplified from gDNA using primer set 27F/1492R (Lane et al. 1985; Fukuda et al. 2016) (Integrated DNA Technologies, Singapore). Reaction mixtures contained 5 µL 5 × Green *Gotaq*^®^ Flexi Buffer (Promega, Madison, WI, USA), 2.5 mM MgCl_2_ (Promega), 0.2 mM each dNTP (Bioline), 10% v/v dimethylsulfoxide (DMSO) (Sigma-Aldrich), 0.4 µM each primer, 0.625 units of *GoTaq*^®^ Hotstart DNA Polymerase (Promega), 3 µL of purified DNA template, and Ultrapure™ water to 25 µL (Invitrogen). Amplification was performed in an MJ Mini™ Thermal Cycler (Bio-Rad, Gladesville, Australia) comprising 94 °C for 2 min, 30 cycles of 94 °C for 30 s, 60 °C for 30 s, 72 °C for 90 s and a final extension at 72 °C for 5 min. PCR amplification was confirmed via gel electrophoresis.

PCR products were submitted to The Ramaciotti Centre for Gene Function Analysis, at UNSW Sydney (NSW, Australia), for purification and preparation for sequencing, using primers 27F and/or 1492R, on the Sanger ABI 3730 Capillary Sequencer (Applied Biosystems, Scoresby, Australia). The resulting sequences (~ 1200 bp) were visualised with FinchTV v1.4.0 trace viewer (Geospiza, Seattle, WA, USA), quality trimmed to ~ 1000 bp and compared with known gene sequences in NCBI GenBank using the BLAST search tool (Altschul et al. 1990). Where strains exhibited homology in cell and colony morphology, as well as 16S rRNA gene sequence identity, they were counted as strains within the same ‘species’ group, with one strain from each group randomly selected as a representative for further analysis (Tables 1 and 2).

**Table 1:**
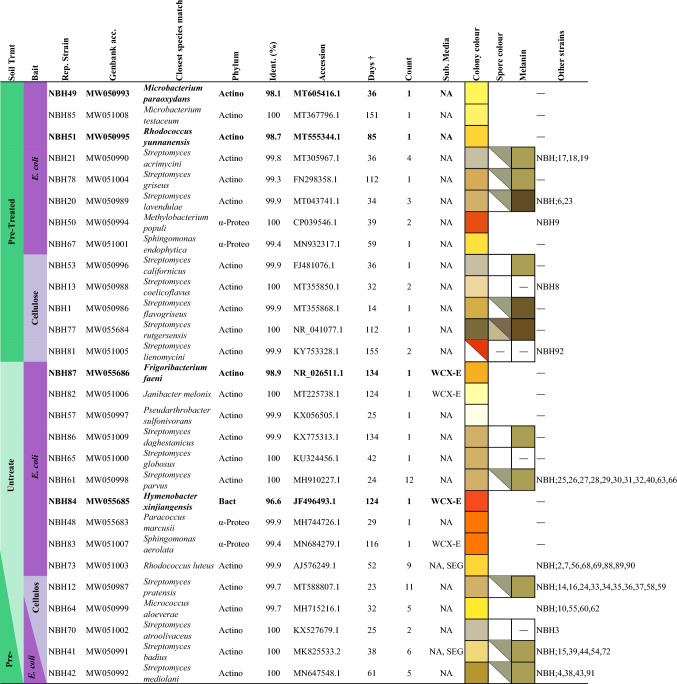
Bacterial strains cultured from Herring Island soil H1/T2/200 by direct soil culturing method (DSC)

^†^Mean days from initial DSC setup to colony picking

Bold indicates strains with < 99% identity to known isolates

Count = number of strains cultivated for this ‘species’ group. NA = 0.75 × nutrient agar. WCX-E = water agar and cycloheximide with *E.coli* bait. SEG = soil extract with gellan gum. Split boxes indicate dual results. ― = none

**Table 2:**
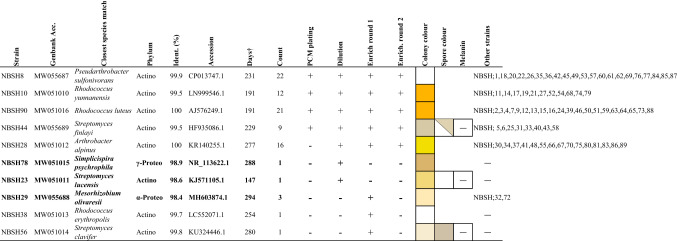
Bacterial isolates cultured from Herring Island soil HI/T2/200 by cold-temperature SSMS method

^†^Mean days from initial SSMS setup to colony picking

Bold indicates strains < 99% identity to known strains

Count = number of strains cultivated for this ‘species’ group. Split boxes indicate dual results. ― none

### Genome analysis for selected strains

Whole genomes were obtained for a selection of the Herring Island isolates as part of a separate study examining BGCs from Antarctic bacteria (Benaud et al. 2021). They included the *Frigoribacterium* sp. NBH87 (GCF_014217665), *Hymenobacter* sp. NBH84 (GCF_014217645), *Mesorhizobium* NBSH29 (GCF_015500055), *Streptomyces* sp. NBSH44 (GCF_014216315) and *Streptomyces* sp. NBH77 (GCF_014216335). Genomic data were used for additional phylogenetic and BGC analyses as described in the following sections.

### Phylogenetic analysis based on 16S rRNA genes for selected strains

Phylogenetic analysis was performed for the strains which exhibited low sequence identity (< 98%) to known species. Near complete 16S rRNA gene sequences were obtained by PCR and Sanger sequencing as previously described for strains *Microbacterium* sp. NBH49 (1368 bp), *Rhodococcus* sp. NBH51 (1241 bp), *Streptomyces* sp. NBSH23 (1374 bp) and *Simplicispira* sp. NBSH78 (1392 bp), while full-length 16S rRNA sequences were obtained for the genome-sequenced strains *Hymenobacter* sp. NBH84 (1497 bp), *Frigoribacterium* sp. NBH87 (1510 bp) and *Mesorhizobium* sp. NBSH29 (1475 bp). Sequences were compared against the EZBiocloud database of validly named species (Yoon et al. 2017), and sequences from the closest 20 matches were downloaded for phylogenetic analysis. Outgroup sequences were selected from members of the same family for each strain. Multiple sequence alignments were performed using MAFFT v7.490 (https://mafft.cbrc.jp/alignment/server/) employing the L-INS-i iterative refinement strategy (Katoh et al. 2019). Aligned sequences were trimmed using trimAl v1.2 with a gap threshold of 0.5 (Capella-Gutiérrez et al. 2009). Phylogeny was inferred by maximum-likelihood method, using the IQ-Tree webserver https://www.hiv.lanl.gov/content/sequence/IQTREE/iqtree.html applying 1000 ultrafast bootstrap iterations, hill-climbing nearest neighbour interchange (NNI) search (Nguyen et al. 2015; Minh et al. 2013), and incorporating additional SH-like approximate likelihood ratio tests (SH-alrt) (Guindon et al. 2010). ModelFinder was used to determine the best-fit phylogenetic model for each tree, which was TN + F + I + G4 for strains NBH87 and NBSH23, TN + F + R2 for NBSH29, TIM + F + R2 for NBH49; TIM2 + F + I + G4 for NBH84 and TIM + F + I + G4 for strains NBH51 and NBSH78 (Kalyaanamoorthy et al. 2017). No sequences failed composition Chi^2^ tests (*p* value < 5%; *df* = 3). The resulting newick files were imported to iTOL v. 6.5.2 (https://itol.embl.de/) for tree visualization (Letunic and Bork 2021). Bootstrap values > 50% were displayed.

### Multi-locus phylogenetic analysis for selected genome-sequenced strains

Multi-locus sequence analysis (MLSA) was performed for three strains representing potentially novel species for which genomic data were available: *Hymenobacter* sp. NBH84 (GCF_014217645), *Frigoribacterium* sp. NBH87 (GCF_014217665) and *Mesorhizobium* sp. NBSH29 (GCF_015500055) (Benaud et al. 2021). Genomic nucleotide sequences were analysed using the AutoMLST online tool (https://automlst.ziemertlab.com/analyze) in de novo mode against related genomes in the curated database, to produce maximum-likelihood trees with additional IQ-TREE ultrafast bootstrap analysis (1000 replicates) including the closest 50 related genomes based on comparisons between up to 100 shared genes (Alanjary et al. 2019). Following examination of results, a re-analysis was performed to incorporate closest related NCBI type strains and representative species from the genome taxonomy database (GTDB) which were not yet included in the AutoMLST database. These additions were: *Frigoribacterium faeni* NBRC 103066^T^ (GCF_007988805) and *Frigoribacterium endophyticum* AS3.20 (GCF_011759585) for the NBH87 strain MLSA; *Mesorhizobium soli* JCM 19897^T^ (GCF_003012705) and *Mesorhizobium zhangyense* CGMCC 1.15528^T^ (GCF_011045115), both of which had been re-designated as *Pseaudaminobacter* genus in the GTDB; *Mesorhizobium loti* DSM 2626^T^ (GCF_003148495), *Mesorhizobium tamadayense* DSM 28320^T^ (GCF_003863365) and *Mesorhizobium australicum* WSM2073^T^ (GCF_000230995) for the NBSH29 MLSA. For the *Hymenobacter* NBH84 MLSA, all closest related *Hymenobacter* genomes in the database were type strains. Up to 22 of the closest related genomes were included in the final analysis for each tree, with the MLSA comprising 92 core genes for the *Hymenobacter* sp. NBH84 analysis, 72 genes for *Frigoribacterium* sp. NBH87 and 86 genes for *Mesorhizobium* sp. NBSH29 (SI Table 3). The resulting newick files were imported into iTOL v 6.5.2 for tree visualization (Letunic and Bork 2021), with bootstrap support values of > 99% displayed.

**Table 3:** Natural product domain amplification and in situ antimicrobial activity for strains isolated in this study

Rep. Strain	Closest cultured representative	PCR		Mean antimicrobial cross-streak activity (mm)				
		PKS	NRPS	*S. aur*	*B. subt*	*E. coli*	*P. aerug*	*C. albic*
NBSH28	*Arthrobacter alpinus*	–	–	0	0	0	0	0
NBH87	*Frigoribacterium faeni*	–	+	0	0	0	0	0
NBH84	*Hymenobacter xinjiangensis*	–	–	0	0	0	0	0
NBH82	*Janibacter melonis*	–	–	0	0	0	0	0
NBSH29	*Mesorhizobium olivaresii*	–	–	0	0	0	0	0
NBH50	*Methylobacterium populi*	–	–	0	0	0	0	0
NBH49	*Microbacterium paraoxydans*	–	–	0	0	0	0	0
NBH85	*Microbacterium testaceum*	+	–	0	0	0	0	0
NBH64	*Micrococcus aloeverae*	–	–	0	0	0	0	0
NBH48	*Paracoccus marcusii*	–	–	^a^	0	0	0	0
NBSH8	*Pseudarthrobacter sulfonivorans*	–	–	^a^	0	0	0	^a^
NBSH38	*Rhodococcus erythropolis*	–	+	0	0	0	0	0
NBH51	*Rhodococcus yunnanensis*	+	+	0	0	0	0	0
NBSH90	*Rhodococcus luteus*	+	+	0	0	0	0	0
NBSH78	*Simplicispira psychrophila*	+	–	0	0	0	0	0
NBH83	*Sphingomonas aerolata*	+	–	0	0	0	0	0
NBH67	*Sphingomonas endophytica*	–	–	0	0	0	0	0
NBH70	*Streptomyces atroolivaceus*	–	+	5 ± 1	3 ± 1	^a^	0	^a^
NBH41	*Streptomyces badius*	–	+	^a^	^a^	0	0	0
NBH53	*Streptomyces californicus*	–	+	^a^	8 ± 2	^a^	0	^a^
NBSH56	*Streptomyces clavifer*	+	–	^a^	^a^	0	0	0
NBH13	*Streptomyces coelicoflavus*	–	+	3 ± 2	1 ± 1	0	1 ± 1	0
NBH1	*Streptomyces flavogriseus*	–	+	8 ± 1	8 ± 2	^a^	^a^	9 ± 1
NBH86	*Streptomyces daghestanicus*	+	+	1 ± 0	3 ± 2	0	0	0
NBSH44	*Streptomyces finlayi*	+	+	1 ± 0	8 ± 1	^a^	0	0
NBH21	*Streptomyces acrimycini*	–	–	0	4 ± 4	0	0	0
NBH65	*Streptomyces globosus*	–	+	0	3 ± 2	0	0	0
NBH77	*Streptomyces rutgersensis*	+	–	3 ± 1	4 ± 1	0	0	2 ± 1
NBH78	*Streptomyces griseus*	+	+	0	^a^	0	0	0
NBH20	*Streptomyces lavendulae*	–	+	4 ± 0	9 ± 1	^a^	^a^	^a^
NBH81	*Streptomyces lienomycini*	+	+	8 ± 3	12 ± 3	0	0	0
NBSH23	*Streptomyces lucensis*	–	+	2 ± 2	5 ± 2	0	^a^	0
NBH61	*Streptomyces parvus*	+	–	0	^a^	0	0	0
NBH42	*Streptomyces mediolani*	–	+	8 ± 3	5 ± 2	^a^	^a^	^a^
NBH12	*Streptomyces pratensis*	+	+	2 ± 1	0	0	0	0

Antimicrobial activity measurement represents mean and standard deviation of three replicates

^a^Pathogen grew less robustly in all replicates compared with control

### Growth temperature range assessment for selected strains

Growth temperature range was assessed for the seven strains representing potentially novel species, isolated by both DSC (*Microbacterium* sp. NBH49, *Rhodococcus* sp. NBH51, *Hymenobacteria* sp. NBH84 and *Frigoribacterium* sp. NBH87) and SSMS methods (*Streptomyces* sp. NBSH23, *Mesorhizobium* sp. NBSH29 and *Simplicispira* sp. NBSH78). Strains were streaked onto nutrient agar and incubated at RT for 7 days. Starter cultures were prepared by inoculating cells from actively growing single colonies into 30 mL nutrient broth to obtain an OD_600_ of 0.05 for each strain. Two control strains were similarly prepared: cold-adapted strain *Sphingopyxis alaskensis* RB2256, isolated from Alaskan seawater but capable of growth at a wide temperature range (5–45 °C) (Ting et al. 2010; Eguchi et al. 1996); and the mesophilic human pathogen *Staphylococcus aureus* ATCC 25923 (Cowan et al. 1954). Nutrient agar plates were prepared in triplicate for each isolate for incubation at five temperatures: 4 ℃, 10 ℃, 20 ℃, 30 ℃ and 40 ℃. Each quarter plate was inoculated with 10 µL of starter culture from the isolate, two positive controls and a negative control (nutrient broth). Plates were incubated for a total of 30 days, with measurements occurring at 1, 2, 3, 5, 7, 9, 12, 16, 23 and 30 days following inoculation. Colonies were photographed and the diameter measured to the the nearest 0.5 mm to calculate mean growth and standard error.

### PCR screening of selected strains for Type I PKS and NRPS domain sequences

Genomic DNA from thirty-five representative strains (Tables 1 and 2) was PCR screened for the presence of Type I PKS and NRPS genes, targeting the conserved KS/AT and AD domains (Ayuso-Sacido and Genilloud 2005). Each 50 μL reaction comprised 10 μL 5X Green *GoTaq*^®^ Flexi Buffer (Promega), 0.2 mM each dNTP, 2.5 mM MgCl_2_, 10% v/v DMSO, 0.8 μM each primer (PKS: K1F/M6R or NRPS: A3F/A7R), 1.25 units of *GoTaq*^®^ Flexi DNA Polymerase (Promega), 18.75 μL Ultrapure™ water and 5 µL purified gDNA. Thermocycler conditions for Type I PKS comprised 94 °C for 2 min, 30 cycles of 94 °C for 30 s, 55 °C for 30 s, 72 °C for 2 min and final extension 72 °C for 5 min; for NRPS: 95 °C for 5 min, 30 cycles of 95 °C for 30 s, 59 °C for 30 s, 72 °C for 4 min and final extension at 72 °C for 10 min. The positive control was purified genomic DNA from *Streptomyces* strain CZ24, positive for both Type I PKS and NRPS genes (van Dorst et al. 2017).

### In situ antimicrobial testing by cross-streak method for selected strains

The thirty-five strain representatives (Tables 1 and 2) were screened for antimicrobial activity in triplicate using the in situ cross-streak agar method (Carvajal 1947; Hopwood 2007; Kamat and Velho-Pereira 2011). This screening assay provides semi-quantitative results. but with the benefit of stimulating NP synthesis through competitive antagonism (Kamat and Velho-Pereira 2011; Balouiri et al. 2016). Strains were inoculated onto nutrient agar (NA) (Oxoid) as a central streak using a sterile 1 µL loop (SI Fig. 12). Plates were incubated at RT for 1–7 days depending on the genus, to allow sufficient growth and production of active compounds. Test pathogens comprised five opportunistic human pathogenic strains commonly utilised in antibiotic sensitivity testing (ATCC 2014). They included a selection of Gram-positive pathogens: *S. aureus* ATCC 25,923 and *Bacillus subtilis* ATCC 11774; Gram-negative pathogens: *E. coli* ATCC 25922 and *Pseudomonas aeruginosa* ATCC 27853; and one fungal pathogen: *Candida albicans* ATCC 10231. Test pathogens were streaked from the edge of the plate towards the polar isolates in perpendicular lines using a 1 µL sterile loop (SI Fig. 12). Plates were incubated for a further 1–4 days at RT, the zone of clearing was measured, and the mean and standard deviations of replicates were recorded. In cases where pathogens grew less robustly in all replicates with comparison to the control, but distinct clearing zones were not observed, an inhibition (a) result was recorded (Table 3). For controls, for which experiments were also conducted in triplicate, negative controls consisted of pathogens streaked in an identical way with no isolate, while positive controls were carried out by disc diffusion method (Bondi et al. 1947), whereby a small portion of test pathogen colony was inoculated into 1 mL phosphate-buffered saline (PBS), spread-plated onto NA and allowed to dry. Discs infused with tobramycin (30 µg/ mL) (Bio-Rad) were applied to the bacterial lawns, while amphotericin B discs (10 µg/ mL) (Sigma-Aldrich) were applied to *C. albicans* lawns and the plates incubated at RT for 48 h before measurement of zones of clearing.

## Results

### Bacterial 16S rRNA gene sequence diversity and selection of Herring Island soil

**Fig. 3:**
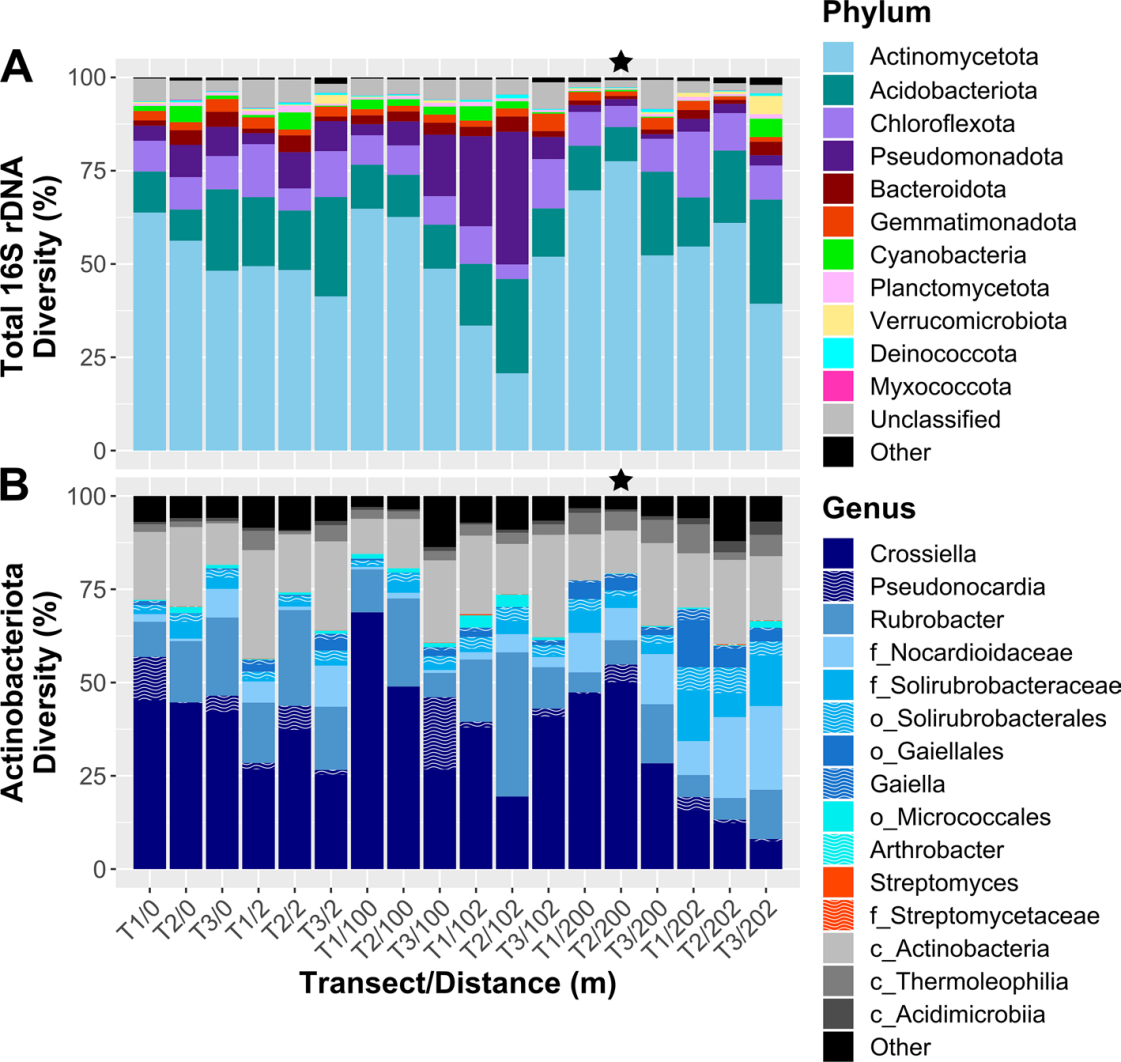
Relative abundance plots of bacterial taxa present in eighteen Herring Island soils, sampled along three parallel 300 m-long transects. **A** Relative abundance of taxa by phylum. **B** Relative abundance of *Actinomycetota* by genera. Where sequences could not be classified to genera level, the taxon level achieved is denoted by the prefix for family, order or class, respectively. Overall, Herring Island soils were dominated by *Actinomycetota*, followed by *Acidobacteriota*, *Chloroflexota* and *Pseudomonadota*. The diversity of the *Actinomycetota* phyla at genera level comprised a high proportion of *Crossiella* (order *Pseudonocardiales*), unclassified *Actinobacteria* class, *Rubrobacter* (order *Rubrobacterales*), and unclassified *Nocardioidaceae* family. *Streptomyces* were detected only in low abundance. Soil T2/200 (denoted by a star) was used for culturing experiments

Taxonomic analysis of amplicon sequencing of the 16S rRNA gene from HI soils (*n* = 18) at phyla level revealed a high relative abundance of *Actinomycetota* (21–78%), followed by *Acidobacteriota* (8–28%), *Pseudomonadota* (1–36%) and *Chloroflexota* (4–18%) (Fig. 3A). The relative abundance of *Myxococcota* was low (0–0.17%). At order level, *Actinomycetota* sequences were dominated by *Pseudonocardiales* (8–69%), *Rubrobacterales* (5–39%), *Propionibacteriales* (1–26%) and *Solirubrobacterales* (2–21%), while relative abundance of *Streptomycetales* was low (< 0.3%). A large proportion (9–29%) of sequences could not be classified beyond class level. Of sequences classifiable to genus level, *Crossiella* (order *Pseudonocardiales*) and *Rubrobacter* (order *Rubrobacterales*) had the highest relative abundance (Fig. 3B, SI Table 2).

The soil selected for culturing experiments, HI/T2/200, was chosen because it had the highest relative abundance of *Actinomycetota* (78%) (Fig. 3A, indicated by star), with the 200 m distance samples found to contain significantly higher *Actinomycetota* (*Q*-value < 0.05) than both the 2 m distance and 102 m distance samples (0.58 and 0.96 log-fold increase respectively) (SI Table 2).

### Summary of total bacterial strains cultured from Herring Island soil

Overall, culturing from the HI/T2/200 soil resulted in a final library of 166 strains assigned to 35 different ‘species' groups (Tables 1 and 2). *Actinomycetota* was the dominant phyla recovered, totaling 156/166 (94%) of strains, comprising the orders *Streptomycetales* 64/166 (38%), *Micrococcales* 48/166 (29%) and *Corynebacteriales* 44/166 (26%). Of the remaining strains, nine were *Pseudomonadota*, from the orders *Rhizobiales* 5/166 (3%), *Rhodobacterales* 1/166 (0.6%), *Sphingomonadales* 2/166 (1%) and *Burkholderiales* 1/166 (0.6%), and one was a *Bacteroidota*, from the order *Cytophagales*. The DSC culturing method produced 79 of the 166 strains, while 87 were isolated using the SSMS (Tables 1 and 2). Strains assigned to three groups related to species *Pseudarthrobacter sulfonivorans* (e.g. NBH57 and NBSH8), *Rhodococcus luteus* (e.g. NBH73 and SH90) and *Rhodococcus yunnanensis* (e.g. NBH51 and NBSH10) were recovered by both DSC and SSMS methods: carotenoid-like pigmented bacteria spanned all three cultured phyla, with 76/166 (46%) isolates displaying variations from pale yellow pigmentation through orange and red (Tables 1 and 2).

### Bacteria isolated from direct soil culturing (DSC) method

**Fig. 4:**
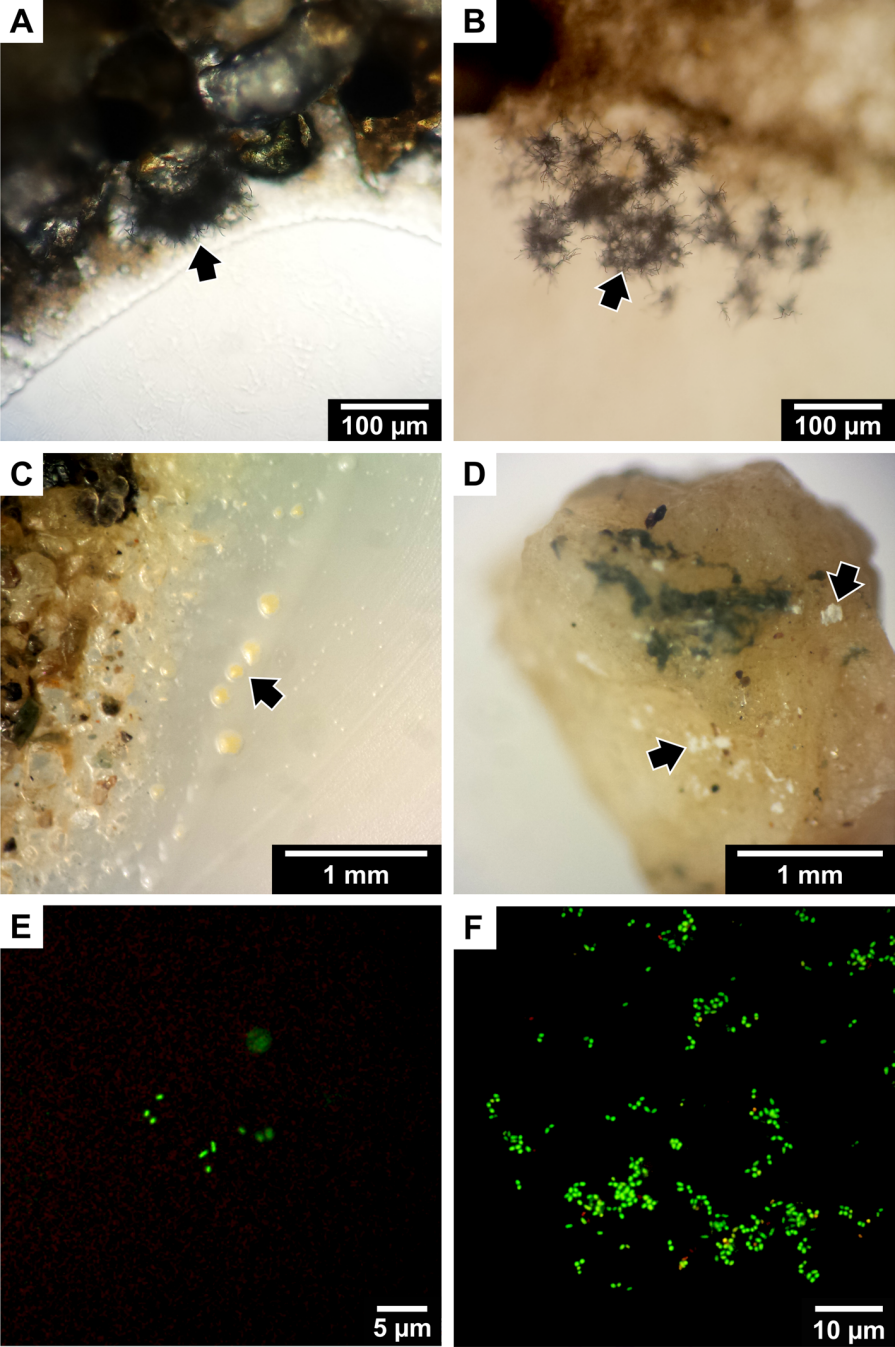
Microscopic images of direct soil cultures (DSC) from Herring Island soil (WCX agar with *E. coli* bait), and epi-fluorescence microscopy of cold-incubated soil substrate membrane system (SSMS) cultivated microcolonies. **A** DSC light microscopy at 9 d incubation showing *Streptomyces* substrate mycelium extending from soil particles. **B** DSC light microscopy at 14 d incubation showing *Streptomyces* mycelium spreading throughout agar. **C** DSC stereomicroscopy at 21 d incubation. Sub-culturing revealed the yellow-pigmented microcolonies to be multi-species which further sub-culturing recovered as *Paracoccus* and *Rhodococcus* spp. **D** DSC stereomicroscopy at 27 days incubation showing sporulating microcolonies atop soil particles. When sub-cultured, these colonies gave rise to diverse *Streptomyces* isolates. **E** SSMS microcolonies visualised using epi-fluorescence microscopy employing the LIVE/DEAD BacLight Bacterial Viability stain whereby live intact cells fluoresce green, and dead/damaged cells fluoresce red. At 50 days incubation, only a few small microcolonies were observed. Cells were cocci and short rods < 1 μm in size. **F** SSMS at 162 days incubation numerous live microcolonies were observed, comprising small cocci and short rod-shaped cells, with cells < 1 μm in size predominating

After 8 days incubation on water agar and cycloheximide (WCX) plates, mycelium-like microcolonies were observed extending out from soil particles and into the surrounding media (Fig. 4A, B). These were revealed as diverse *Streptomyces* strains following sub-culturing and 16S rRNA gene sequencing (Table 1). Over an ~ 8 month extended incubation period, visible colonies were continuously sub-cultured from the surfaces of the agar and soil particles (Fig. 4C, D). *Myxococcota* fruiting bodies were not detected during the observation period. DSC culturing led to the recovery of a total of 79 bacterial strains, assigned to 28 different ‘species’ groups (Table 1). The formation of detectable colonies took longer than 1 month for 51/79 (65%) of the strains (Table 1).

The *E. coli* lawn method uniquely yielded 10 of the recovered genera (*Microbacterium*, *Methylobacterium*, *Sphingomonas*, *Frigoribacterium*, *Janibacter*, *Pseudarthrobacter*, *Hymenobacter*, *Paracoccus*, *Rhodococcus* and one of the *Streptomyces* groups), while the cellulose baiting method uniquely yielded six of the *Streptomyces* groups (represented by strains NBH53, NBH13, NBH1, NBH12, NBH77 and NBH81). A further three *Streptomyces* groups and one *Micrococcus* sp. were common to both cellulose and *E. coli* baiting methods (represented by strains NBH70, NBH41, NBH42 and NBH64) (Table 1). Soil samples which had been pre-treated with heat and sonication uniquely led to members of 13 ‘species’ groups comprising five genera (e.g. *Microbacterium* sp. NBH49, *Rhodococcus* sp. NBH51, *Streptomyces* sp. NBH21, *Methylobacterium* sp. NBH50, *Sphingomonas* NBH67, while nine groups comprising seven genera were recovered only from untreated soil samples (e.g. *Frigoribacterium* NBH87, *Janibacter* sp. NBH82, *Pseudarthrobacter* sp. NBH57, *Streptomyces* sp. NBH86, *Hymenobacter* sp. NBH84, *Paracoccus* sp. NBH48 and *Sphingomonas* sp. NBH83) (Table 1). The remaining six ‘species’ groups (three genera) were recovered from both soil preparations.

The majority of strains (73/79, 92%) recovered by DSC were *Actinomycetota*, with most belonging to the *Streptomyces* genus (53/79, 67%) (Fig. 4, Table 1). The remaining strains were *Alphaproteobacteria* (five strains) and one representative from the *Bacteroidota* phylum. Isolates were predominantly recovered from secondary cultivation on 0.75 × nutrient agar (NA), rather than WCX or soil extract with gellan gum plates (SEG). Those recovered from NA sub-cultures were *Methylobacterium*, *Microbacterium*, *Micrococcus*, *Paracoccus*, *Pseudarthrobacter*, *Rhodococcus*, *Sphingomonas* and *Streptomyces* spp., while *Janibacter* sp. NBH82, *Sphingomonas* sp. NBH83, *Hymenobacter* sp. NBH84 and *Frigoribacterium* sp. NBH87 were retrieved through secondary cultivation on WCX/*E. coli* plates (Table 1). Strains assigned to two ‘species’ groups, *R. luteus* and *S. badius*, were recovered from both NA and SEG sub-culture media.

The median time taken from initial DSC setup to visible colony formation was 40 days (Table 1). Strains which were particularly slow growing or had lengthy lag phases (taking > 100 days for visible growth to appear) included *Frigoribacterium* sp. NBH87, *Janibacter* sp. NBH82, *Sphingomonas* sp. NBH83 and *Hymenobacter* sp. NBH84, as well as several of the morphologically striking *Streptomyces* spp.; NBH77 and NBH81 (Table 1, SI Fig. 2).

Most DSC isolates exhibited high 16S rRNA gene sequence identity to known bacterial species (99–100%) based on single-end sequence reads ~ 900 bp. Only four strains exhibited < 99% similarity to their closest cultured representatives, which was confirmed by obtaining near-complete sequences (~ 1400 bp); these were *Frigoribacterium* sp*.* NBH87 (98.9%) and *Hymenobacter* sp. NBH84 (96.6%) which were recovered from secondary cultivation on WCX *E. coli*; and *Microbacterium* sp. NBH49 (98.1%) and *Rhodococcus* sp. NBH51 (98.7%), both recovered from NA sub-culture (Table 1). Amongst the 15 *Streptomyces* spp. isolated, sporulation pigments comprised olive green, white and brown, while colony morphology predominantly spanned grey, tan and brown (Table 1, SI Fig. 2). One strain, *Streptomyces* sp. NBH81, produced a striking red colony pigmentation (SI Fig. 2E). Diffused melanin-like pigments ranged from dark brown, as in *Streptomyces* sp. NBH20 and *Streptomyces* sp. NBH77 (SI Fig. 2A, F), to tan-coloured, for example *Streptomyces* sp. NBH21 and *Streptomyces* sp. NBH61 (SI Fig. 2B). Strains that produced no diffuse melanin-like pigments included *Streptomyces* sp. NBH70 and *Streptomyces* sp. NBH81 (SI Fig. 2C, E).

### Bacteria recovered from cold-temperature SSMS method

At 50 and 78 days incubation using the SSMS at 4 °C, small microcolonies comprising three or more small cocci or short rod-shaped cells < 1 µm in size were visualised using epi-fluorescent microscopy (Fig. 4E), with no red fluorescing (damaged) cells detected. After 162 days of incubation, larger microcolonies were present, predominantly composed of green-fluorescing cocci and short rod-shaped cells, with a small number of red fluorescing dead or damaged cells also observed (Fig. 4F). Occasional rod-shaped cells (6–8 µm) were also present at 162 days.

Secondary cultivation from the SSMS onto RTSV media produced bacterial communities dominated by three main morphotypes: large white, large yellow or small orange-coloured colonies (SI Fig. 3A), all of which were Gram-positive coryneform bacteria. These morphotypes were later identified by 16S rRNA gene sequencing as *Pseudarthrobacter* (SI Fig. 3B), *Arthrobacter* (SI Fig. 3E) and *Rhodococcus* spp. (SI Fig. 3C) respectively. In total, 87 pure cultured strains were obtained from the cold-temperature SSMS, which were taxonomically assigned to 10 different ‘species’ groups (Table 2). The SSMS isolated strains were predominantly *Actinomycetota* (83/87, 95%), including *Arthrobacter*, *Pseudarthrobacter*, *Rhodococcus* and *Streptomyces* genera, followed by two *Pseudomonadota* genera, *Mesorhizobium* and *Simplicispira* (Table 2).

Of the three secondary cultivation methods employed, mixed community enrichment resulted in the recovery of strains from eight different ‘species’ groups (Table 2) and uniquely led to the recovery of three strains: *Rhodococcus* sp. NBSH38, *Mesorhizobium* sp. NBSH29 and *Streptomyces* sp. NBSH56, all of which were only recovered through one enrichment round (Table 2). The dilution-plating method resulted in seven ‘species’ groups, uniquely including *Streptomyces* sp. NBSH23 and *Simplicispira* sp. NBSH78 (Table 2), while PCM plating produced strains from only four ‘species’ groups: *Rhodococcus* sp. NBSH10, *Rhodococcus* sp. NBSH90, *Pseudarthrobacter* sp. NBSH8 and *Streptomyces* sp. NBSH44, with strains recovered from all cultivation conditions (Table 2).

Cold-incubated SSMS isolates were slow to grow, taking a median of 280 days from initial SSMS setup to visible colony formation on RTSV plates (Table 2). Sub-culturing from the 50 days incubated membranes led to the recovery of only five strains, belonging to *Rhodococcus* and *Pseudarthrobacter*, with the majority appearing following additional rounds of enrichment. In contrast, secondary cultivation attempts from the 78 days and 162 days incubated membranes led to recovery of 30 and 52 strains, respectively.

Interestingly, the *Streptomyces* sp. NBSH23 strain grew on RTSV only in co-culture with other microorganisms, suggesting helper strains were supplying nutritional requirements not provided by the low-nutrient media alone. Pure colony isolation of this strain was only achieved through sub-culture onto 0.75 × nutrient agar (NA). All cold-adapted species cultured by the SSMS at 8 °C were also capable of later growth at RT and on 0.75 × NA.

Three SSMS-cultivated strains had < 99% 16S rRNA gene sequence identity to previously cultured strains: *Streptomyces* sp. NBSH23, *Mesorhizobium* sp. NBSH29 strain and *Simplicispira* sp. NBSH78 (Table 2, SI Fig. 3F). The remaining seven SSMS-isolated taxa were 99 – 100% similar to previously characterised bacteria.

### Phylogenetic analysis based on 16S rRNA sequences for seven strains

Maximum-likelihood phylogenetic trees confirmed that the seven low-identity strains diverged from their most closely related type species (SI Figs. 4–10). For *Microbacterium* sp. NBH49 (SI Fig. 4), the closest relationship was seen with *Microbacterium foliorum* DSM 12966^T^ with branch support of 87%, within a clade which also included *Microbacterium natoriense* TNJL143-2^T^. *Rhodococcus* sp. NBH51 (SI Fig. 5) formed a distinct branch with robust bootstrap support (100%), cladding with *R. yunnanensis* NBRC 103083^T^, *Rhodococcus sovatensis* H004^T^ and *Rhodococcus fascians* LMG 3623^T^. A distinct branch with strong support was similarly observed for *Hymenobacter* sp. NBH84 (SI Fig. 6), forming a clade with *Hymenobacter defluvii* POA9^T^ and *Hymenobacter profundi* M2^T^*. Frigoribacterium* sp. NBH87 (SI Fig. 7) formed a distinct branch within a clade containing the only three *Frigoribacterium* type strains, *Frigoribacterium faeni* NBRC 103066^T^*, Frigoribacterium salinisoli* LAM9155^T^ and *F. endophyticum* EGI 6500707^T^, though bootstrap support was low (39%). *Streptomyces* sp. NBSH23 (SI Fig. 8) formed a long branch with low bootstrap support (54%) within a clade containing *Streptomyces adustus* WH-9^T^, *Streptomyces yokosukanensis* DSM 40224^T^ and *Streptomyces griseochromogenes* ATCC 14511^T^, with the closest relationship to *Streptomyces albosporeus* subsp. *labilomyceticus* NBRC 15387^T^. *Mesorhizobium* sp. NBSH29 did not show close relationship to other *Mesorhizobium* type strains, branching deeply within a strongly supported clade (100%) containing *Mesorhizobium chacoense* PR5^T^ and *Mesorhizobium olivaresii* CPS13^T^ (SI Fig. 9). *Simplicispira* sp. NBSH78 was related most closely to *Simplicispira psychrophila* DSM 11588^T^, forming a distinct branch with moderate branch support (74%) (SI Fig. 10) within a clade including *Simplicispira piscis* RSG39^T^, *Simplicispira limi* EMB325^T^, *Simplicispira suum* SC1-8^T^ and *Simplicispira metamorpha* DSM 1837^T^.

### Multi-locus phylogenetic analysis for selected strains

**Fig. 5:**
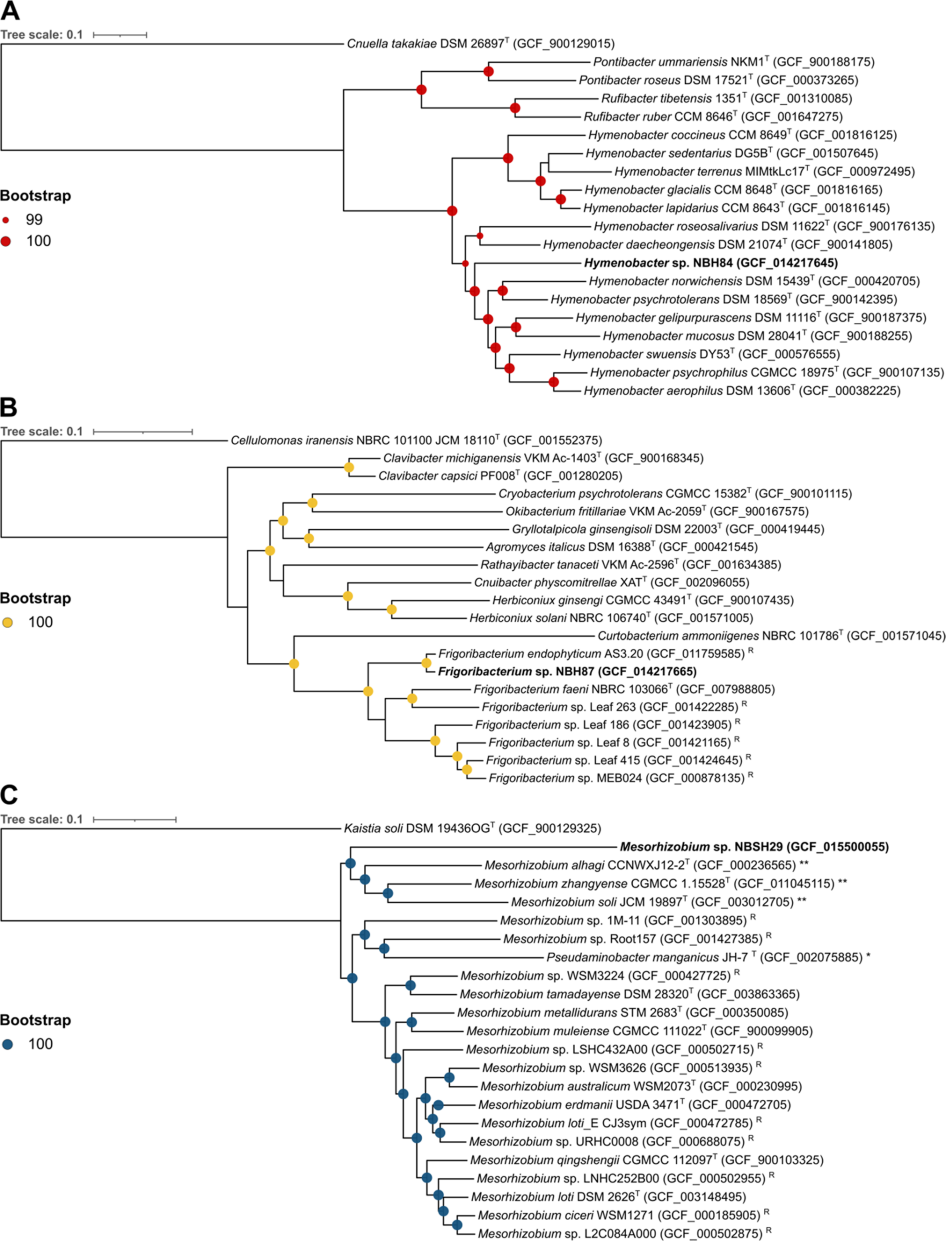
Multi-locus phylogenies of genomes from three Herring Island bacteria, analysed using AutoMLST de novo workflow, compared against closest related genomes of type strains or GTDB representative species. **A** *Hymenobacter* sp. NBH84. The outgroup species was *Cnuella takakiae* DSM 26897^T^ with 92 core genes used for comparison. **B** *Frigoribacterium* sp. NBH87. Outgroup was *Cellulomonas iranensis* NBRC 101100^T^. The AutoMLST database contained no *Frigoribacterium* type strains; thus, the closest *Frigoribacterium* genomes depicted are GTDB representative species, with 72 core genes compared. **C** *Mesorhizobium* sp. NBSH29. Outgroup was *Kaistia soli* DSM 19436^T^, with 86 core genes included in the multi-locus analysis. Coloured circles at nodes represent bootstrap support based on 1000 replications, with those ≥ 99% shown. Scale bars represent 0.1 substitutions per site. **Taxonomy for these strains has been re-designated by curators of the GTDB from *Mesorhizobium* to *Pseudaminobacter* genus. *Taxonomy for this strain has been re-designated by the GTDB from *Pseudaminobacter* to *Mesorhizobium* genus. ^T^Indicates type strains. ^R^ indicates GTDB representative species

Genomes for three of the low-identity strains, *Hymenobacter* sp. NBH84, *Frigoribacterium* sp. NBH84 and *Mesorhizobium* sp. NBSH29, were available following sequencing for a separate study (Benaud et al. 2021). In multi-locus sequence analysis, the suggested cutoff for species delineation is an average nucleotide identity (ANI) of ≥ 95% (Alanjary et al. 2019). For *Hymenobacter* sp. NBH84, the closest related type strain was *Hymenobacter psychrotolerans* DSM 18569^T^ (GCF_900142395) with an ANI of 84.4% (SI Table 3), indicating that NBH84 is a distinct species. In phylogenetic analysis, NBH84 formed a distinct branch with robust bootstrap support (100%) (Fig. 5A), within a larger clade of seven *Hymenobacter* spp., which included *H. psychrotolerans* and another cold-adapted strain *Hymenobacter psychrophilus* CGMCC 18975^T^ (Zhang et al. 2008, 2011). For *Frigoribacterium* sp. NBH87, the closest GTDB representative species was *Frigoribacterium* sp. MEB024 (GCF_000878135), with an ANI of 86.2% (SI Table 3); however, a closer relative was identified as *Frigoribacterium* sp. Leaf 164, a non-representative strain, with an ANI of 98.6%. A GTDB representative species was identified as a close relative to the Leaf 164 strain: *F. endophyticum* AS3.20 (GCF_011759585) (Wang et al. 2015), and this strain was subsequently included in a second MLSA. Phylogenetic analysis confirmed this relationship, with the NBH87 strain forming a clade with *F. endophyticum* (Fig. 5B). Branches showed strong bootstrap support (100%), and with an ANI > 95%, results indicate that NBH87 and Leaf 164 are both *F. endophyticum* strains (SI Table 3). For *Mesorhizobium* sp. NBSH29, the closest related species in MLSA was *Mesorhizobium qingshengii* CGMCC 112097^T^ (GCF_900103325) with a low ANI of 75.5%, followed by *Mesorhizobium alhagi* CCNWXJ12-2^T^ (GCF_000236565) (ANI 75.3%). Interestingly, in the phylogenetic analysis, NBSH29 formed a distinct clade together with three *Mesorhizobium* strains more recently proposed as members of genus *Pseudaminobacter* by the curators of the GTDB, namely, *M. zhangyense* CGMCC 1.15528^T^ (GCF_011045115), *M. alhagi*^T^ CCNWXJ12-2^T^ (GCF_000236565) and *M. soli*^T^ JCM 19897^T^ (GCF_003012705) (indicated by ** in Fig. 5C). The NBSH29 strain formed a long single branch, diverging by 0.32 substitutions per site, with strong bootstrap support (100%). These results suggest that NBSH29 may be a novel member of genus *Pseudaminobacter*; however, previous authors have reported inconclusive taxonomic placement of members from *Mesorhizobium* and *Pseudaminobacter* genera within the *Phyllobacteriaceae* family, with greater resolution expected as a result of increased genome availability (Kämpfer et al. 1999; Hördt et al. 2020).

### Growth temperature range for selected strains

**Fig. 6:**
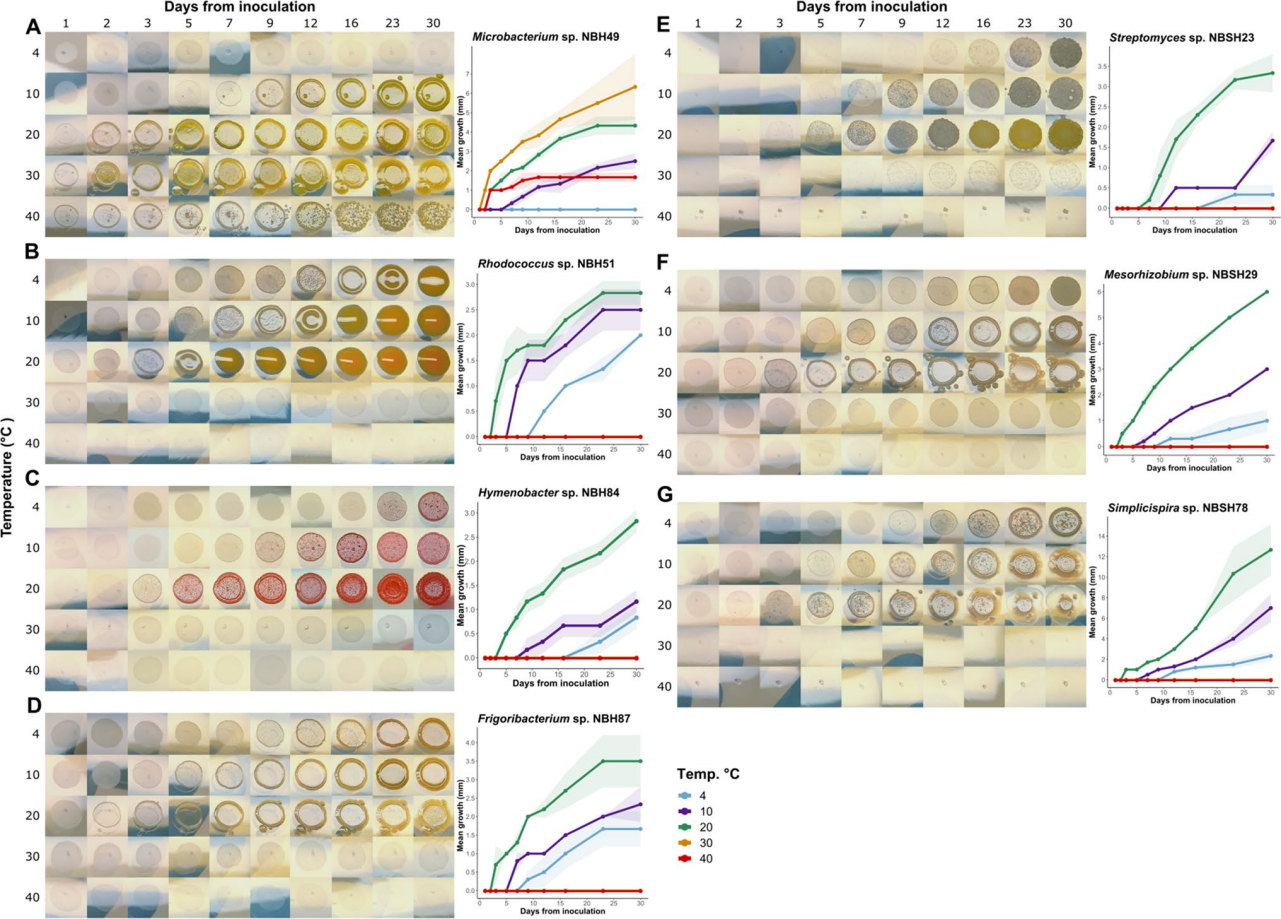
Growth profiles for seven Herring Island bacterial strains, incubated on nutrient agar at five different temperatures: 4, 10, 20, 30 and 40 ℃. Colony diameters were measured at 10 time points between 1 and 30 days, with plots depicting the mean growth for triplicates (lines) and standard error (shaded area). Photographs depict colony morphology at the corresponding time points (size not to scale). **A** *Microbacterium* sp. NBH49, **B** *Rhodococcus* sp. NBH51, **C** *Hymenobacter* sp. NBH84, **D** *Frigoribacterium* sp. NBH87, **E** *Streptomyces* sp. NBSH23, **F** *Mesorhizobium* sp. NBSH29, **G** *Simplicispira* sp. NBSH78

Only one of the seven Antarctic bacterial strains tested; *Microbacterium* sp. NBH49, exhibited growth within the mesophilic temperature range of 10–40 °C (Fig. 6A). NBH49 grew well at 20 °C, optimally at 30 °C, and minimally at 40 °C, while at 10 °C growth was greater than that observed for both controls *S. aureus* and *S. alaskensis* (SI Fig. 11)*.* Along with the two control strains, NBH49 was not observed to grow at 4 °C during the 30 days reporting period. The remaining six Antarctic strains grew within psychrotrophic growth ranges 4–20 °C, with optimal growth at 20 °C and no visible colony growth ≥ 30 °C (Fig. 6B–G). These were strains *Rhodococcus* sp. NBH51 (Fig. 6B), *Hymenobacter* NBH84 (Fig. 6C), *Frigoribacterium* sp. NBH87 (Fig. 6D), *Streptomyces* sp. NBSH23 (Fig. 6E), *Mesorhizobium* sp. NBSH29 (Fig. 6F) and *Simplicispira* sp. NBSH78 (Fig. 6G). Lag times ranged from 5 to 16 days prior to visible growth at 4 °C (mean 11.3 days), 3–7 days at 10 °C (mean 5 days; mesophile controls 8 days) and 2–5 days at 20 ℃ (mean 3.2 days; controls 1.5 days) (Fig. 6, SI Fig. 11). For *Streptomyces* sp. NBSH23, morphology varied at different incubation temperatures, with sporulation observed only at 10 °C and yellow pigmentation developing for the 20 °C-incubated colonies after 16 days (Fig. 6E). *Simplicispira* sp. NBSH78 displayed motility at both 10 and 20 °C (Fig. 6G).

### Natural product domain amplification and in situ antimicrobial activity for selected strains

Type I PKS KS/AT domains were detected in 13/35 (37%) of the selected strains, and 18/35 (51%) were positive for NRPS AD domain PCR (Table 3). Of these, seven were positive for both the Type I PKS KS/AT and NRPS AD domains (Table 3). In the antimicrobial assay, the Gram-positive pathogens were the most commonly inhibited, with 13/35 strains showing zones of clearing for *B. subtilis* and 11/35 for *S. aureus* (Table 3, SI Fig. 12A, B and D). Two of the 35 strains showed clearing against the yeast *C. albicans*, and 1/35 was active against the Gram-negative pathogen *P. aeruginosa* (Table 3). *Streptomyces* was the only genus that produced zones of clearing, although some inhibition of pathogen growth was evident for *Paracoccus*, *Pseudarthrobacter* and *Rhodococcus* genera (Table 3, SI Fig. 12). Three strains showed inhibitory activity against all five pathogens examined: *Streptomyces* spp. NBH1, NBH20 and NBH42, while fifteen strains (43%) showed no activity against the pathogens tested here (SI Fig. 12C).

## Discussion

### Bacterial diversity of Herring Island

Both *Actinomycetota* and *Myxococcota* were targeted here using two unconventional culturing techniques, DSC and SSMS, which utilise the microorganisms originating from soil as a substrate for growth (Ferrari et al. 2008; Shimkets et al. 2006). While no *Myxococcota* were recovered, DSC and SSMS were effective culturing techniques for the capture of diverse genera from Antarctic soil, resulting in a total library of 166 strains, spanning 35 bacterial species across 14 genera and three phyla. The goal to target *Myxococcota* by DSC was optimistic, as they are known to be predominantly mesophilic, with only four psychrophilic members recorded (Ruckert 1985; Shimkets et al. 2006; Brockman and Boyd 1963; Dawid et al. 1988). Furthermore, our bacterial community amplicon sequencing indicated a low relative abundance of the phylum in the HI soil (0.03%). Sequencing showed that HI soils support a remarkably high relative abundance of *Actinomycetota*, up to 78%. This is comparable with hyper-arid soils such as Antarctica’s McMurdo Dry Valleys and Chile’s Atacama desert, which report 73% and 88% *Actinomycetota*, respectively (Lee et al. 2012; Crits-Christoph et al. 2013). Globally, both cold and hot deserts consistently host a high proportion of *Actinomycetota*, typically between 20 and 40% (Neilson et al. 2012; Makhalanyane et al. 2015; Cary et al. 2010), compared with 5–20% for non-arid soil biomes (Fierer et al. 2012; Baldrian et al. 2012; Janssen 2006). The dominance of *Actinomycetota* in the HI soil sequence data was also reflected in our cultured libraries, comprising 94% of all isolates recovered, and to our knowledge this is the first report isolating a substantial number of *Actinomycetia* using either DSC or SSMS methods. At lower taxonomic levels, cultured *Actinomycetota* diverged from amplicon-sequenced communities, with 38% of cultured isolates belonging to *Streptomyces* species, despite *Streptomycetales* being undetected in amplicon sequencing for the soil. This result likely indicates the more readily culturable nature of *Streptomyces* when compared to the rarely isolated genera *Crossiella* and *Rubrobacter* which dominated our amplicon sequencing data (Dhakal et al. 2017; Qin et al. 2009; Floyd et al. 2005); therefore DSC and SSMS methods did not select for these rare *Actinomycetota* genera. We also note that nearly 12% of the *Actinomycetota* sequences were unclassifiable to lower taxonomic levels. An overrepresentation of spore formers such as *Streptomyces* in culture collections has been observed previously (Floyd et al. 2005). Additionally, *Streptomyces* are known to exhibit resistance to cell lysis which can make DNA extraction problematic (Kutchma et al. 1998), and some authors suggest that the genus may subsist in soil predominantly as dormant, resilient spores (Mayfield et al. 1972). Our amplicon sequencing data may thus have underestimated *Streptomyces*, or introduced bias via the chosen sequencing platform or primer set (Janssen 2006). Our culturing methods were successful at retrieving both mycelial (i.e. *Streptomyces*) and amycelial *Actinomycetota* (i.e. family *Micrococcaceae*, e.g. *Arthrobacter* and *Micrococcus*; family *Microbacteraceae*, e.g. *Frigoribacterium* and *Microbacterium* spp.; and family *Dermatophilaceae*; *Janibacter*).

*Pseudomonadota* and *Bacteroidota* were of relatively low abundance in our amplicon sequencing data (1.8% and 0.9%), and these phyla represented 5% and 0.6%, respectively, in culturing efforts. The *Actinomycetota*, *Pseudomonadota*, *Bacillota* and *Bacteroidota* phyla have traditionally dominated cultured bacterial libraries from diverse environments, including Antarctic soils (Floyd et al. 2005; Janssen 2006; Tanaka et al. 2017; Wong et al. 2019; Pudasaini et al. 2017). Commonly isolated genera from East Antarctic soils include *Arthrobacter*, *Rhodococcus*, *Hymenobacter*, *Methylobacterium*, *Micrococcus*, *Sphingomonas* and *Streptomyces* (Lambrechts et al. 2019; Peeters et al. 2011; Wong et al. 2019; Pudasaini et al. 2017), and our results are consistent, highlighting that these taxa are well adapted to survive the challenging environmental conditions. Here, we report a higher diversity of *Streptomyces* strains than previous Antarctic soil culturing efforts (Pulschen et al. 2017; Rego et al. 2019; Pudasaini et al. 2017). Though these results are not directly comparable, our findings suggest that DSC may be a useful tool for the isolation of *Streptomyces* spp., and further, that the pre-treatment of soil by heat and sonication was valuable, with a greater diversity of *Streptomyces* species originating from pre-treated soil DSC cultures. Interestingly, during the DSC methods employed here, *Streptomyces* microcolonies were observed growing on rock particles and were conspicuous as white microcolonies atop mineral surfaces using stereomicroscopy. Mineral substrates may thus have been supporting the bacteria’s nutritional requirements, encouraging future work to examine HI lithobiontic communities. In both cold and hot deserts, lithic substrates are known to be an important ecological niche, harbouring microbial communities which are provided protection from environmental stressors such as high UV radiation, fluctuating temperatures, strong winds and desiccation (Pointing and Belnap 2012; Le et al. 2016). Further, lithobiontic communities are involved in nutrient cycling and rock weathering processes and are thought to significantly contribute to the function and ecology of Antarctic soils (Abdulla 2009; Le et al. 2016; Pointing and Belnap 2012; León-Sobrino et al. 2019).

Carotenoid-like pigmentation has been reported to be widespread in cold-adapted microorganisms (Baraúna et al. 2017; De Maayer et al. 2014; Koblížek and Brussaard 2015; Peeters et al. 2011) and this was similarly found here with 46% of recovered strains displaying yellow to red pigmentation. These comprised members of *Arthrobacter* (e.g. NBSH28), *Frigoribacterium* (NBH87), *Hymenobacter* (NBH84), *Janibacter* (NBH82), *Methylobacterium* (e.g. NBH50), *Microbacterium* (e.g. NBH49, NBH85), *Micrococcus* (e.g. NBH64), *Paracoccus* (NBH48), *Rhodococcus* (e.g. NBH51, NBSH90), *Sphingomonas* (e.g. NBH67, NBH83) and *Streptomyces* (NBH81). Carotenoids are most commonly associated with protection from UV radiation via the scavenging of free radicals such as singlet oxygen (Walter and Strack 2011; Maresca et al. 2008), but they are also hypothesised to assist with homeoviscous adaptation, playing a regulatory role in membrane fluidity (Chattopadhyay and Jagannadham 2001; Walter and Strack 2011). Furthermore, carotenoids function as accessory light-harvesting pigments in aerobic anoxygenic phototrophs (AAP), assisting in bacteriochlorophyll-mediated photosynthesis (Tahon and Willems 2017; Imhoff et al. 2018; Koblížek and Brussaard 2015). AAP comprise certain members of *Alpha*- *Beta*- and *Gammaproteobacteria* and include a number of genera which are commonly recovered from polar soils, and which were also isolated here, such as *Methylobacterium* and *Sphingomonas* (Makhalanyane et al. 2015; Tahon and Willems 2017; Walter and Strack 2011; Imhoff et al. 2018).

### Isolating cold-adapted strains

Previously, the soil substrate membrane system (SSMS) has been used for the capture of new members from *Pseudomonadota*, *Actinomycetota*, *Bacillota* and *Bacteroidota* from diverse environments including temperate garden soils (Ferrari et al. 2005), heavy metal-contaminated acid mine drainages (Delavat et al. 2012), pristine and fuel-spiked polar soils (van Dorst et al. 2016), and polycyclic aromatic hydrocarbon-contaminated oil fields (Zhao et al. 2017). Here, we adapted the SSMS for the isolation of cold-adapted bacteria using < 8 °C incubation conditions. While a lower diversity of taxa was obtained from SSMS in comparison to DSC for the same HI soil, 70% of the strains isolated from SSMS were not recovered by DSC; thus, we increased our inventory of cold-adapted species for further analysis. All isolates retrieved by the cold-incubated SSMS were capable of growth at 21 °C, indicating that they are not obligate psychrophiles. During dark winter months, East Antarctic surface soil temperatures are known to reach − 20 °C (McWatters et al. 2016), while in summer temperatures up to + 18 °C have been recorded, along with large daily fluctuations (~ 10 °C) correlating with the incoming solar radiation (Aislabie et al. 2004; Balks et al. 2002). Previous culturing studies have similarly noted a tendency towards psychrotrophy in terrestrial Antarctic microorganisms, attributed to the regular freeze–thaw cycles endured by the resident microbes (Morita 1975; De Maayer et al. 2014; Soina et al. 2004). Where growth temperature range was assessed, both SSMS and DSC-isolated bacteria grew actively within the psychrophilic range of 4–20℃, with optimal growth at 20℃. The exception was *Microbacterium* sp. NBH49, which exhibited a mesophilic growth range. This is comparable with the closely related *Microbacterium* type strains such as *M. foliorium* and *M. paraoxydans* which grow optimally between 25 and 37 ℃ (Behrendt et al. 2001; Laffineur et al. 2003).

### Extended incubation times led to recovery of novel and rarely cultured species

For improved capture of novel and rare soil bacteria, solid media cultivation has been shown to be superior to liquid media enrichment (Schoenborn et al. 2004; Janssen et al. 2002). In liquid media, the faster growing members of the microbial community out-compete slower growing or less abundant members, while solid media provides spatial separation, minimising competition for resources, as well as interaction with undesirable components such as antibiotics, lysing agents or bacteriophages (Schoenborn et al. 2004). For the SSMS, microcolonies are first cultivated on the solid surface of the PCM, but the successful transfer of total diversity enriched by this method onto artificial media for macrocolony purification continues to be a challenge (van Dorst et al. 2016). Here, we found benefit to further enrichment of the PCM community in liquid media prior to plating onto solid media, resulting in the greatest number and diversity of recovered strains, in addition to capture of the potentially novel species *Mesorhizobium* sp. NBSH29. Further enrichments also extended overall incubation times and extended incubation times are known to assist in recovery of rare, oligotrophic taxa, especially those from nutrient poor environments, and soils where the communities are largely dormant (Pulschen et al. 2017; Alain and Querellou 2009; Davis et al. 2005; Janssen et al. 2002). Here, extended incubation times (> 100 days) led to the recovery of several strains with lower similarity to those known (97–98% identity), for example, *Hymenobacter* sp. NBH84 and *Mesorhizobium* sp. NBSH29, both of which are likely to be novel species according to multi-locus sequence analysis. Additionally, morphologically distinct *Streptomyces* strains such as NBH77 and NBH81 were also recovered at up to 5-month incubation times. The expression of pigments in bacteria often coincides with nutrient deprivation (Couso et al. 2012; Liu et al. 2013). Thus, lengthy culturing times on oligotrophic substrates may have assisted in the visible differentiation and isolation of diverse species. Other slow-growing strains included the rarely isolated *Actinomycetota* genera, *Frigoribacterium* and *Janibacter* (Tiwari and Gupta 2013; Kampfer et al. 2000; Martin et al. 1997). Secondary cultivation on richer media led to faster growth, as seen in the temperature profile assays, where growth was observed ~ 3 days at 20 °C, compared with 67 days for original colonies picked from DSC cultures; and an average of ~ 11 days for 4 °C incubation, compared with > 200 days at < 10 °C for SSMS cultures. While this shows an ability to increase cell division in nutrient-rich conditions, Antarctic strains were still slower to grow on average than control mesophiles, indicative of oligotrophs accustomed to a low nutrient environment (Fierer et al. 2007).

### Bioactive natural product capacity of Herring Island soil bacteria

*Actinomycetota* is traditionally one of the four main phyla commonly associated with NP biosynthesis, along with *Pseudomonadota*, *Cyanobacteria* and *Bacillota*, and the *Streptomyces* have long been considered genetically and chemically ‘gifted’ in terms of antibiotic NP synthesis, producing 68 of 100 antibiotics deemed most clinically important (Baltz 2017; Katz and Baltz 2016; Bérdy 2005). *Streptomyces* are known to be non-obligate epibiotic predators, capable of lysing their prey via secretion of antimicrobial compounds and hydrolysing enzymes (Kumbhar et al. 2014; Pérez et al. 2011, 2016). Accordingly, the *Streptomyces* spp. from our cultured library showed the greatest antibiotic NP potential, displaying the strongest clearing of pathogen growth. Of the Antarctic *Streptomyces* isolated here, we consider the most promising strains for future chemical analysis work to be those which inhibited the growth of multiple pathogens, particularly against Gram-negative pathogens which are of particular concern in terms of antimicrobial resistance (WHO 2014). These strains were NBH1, NBH13, NBH77, NBH81, NBH42, NBH20 and NBSH44. Horizontal gene transfer and homologous recombination has been found to be widespread amongst *Streptomyces* spp.; thus, bioactive compounds have the potential to be commonly produced amongst certain lineages, or secreted uniquely by individual strains (Jorgensen et al. 2009; James and Daniel 2010). While further work is required to characterise the compounds produced by the Antarctic strains isolated here, related *Streptomyces* species, such as *S. lavendulae*, *S. coelicoflavus* and *S. flavogriseus*, have been shown to produce compounds with broad-spectrum antimicrobial activity, including minimycin, showdomycin and lavencidin in *S. lavendulae*; BC01-C1, C2 and C3 in *S. coelicoflavus*; and actinomycin and holomycin in *S. flavogriseus* (Raghava Rao et al. 2017; Yoshioka et al. 2021; Wei et al. 2017), which may be relevant for activity found in our related strains NBH20, NBH13 and NBH1. It is known from genomic analyses that *Streptomyces* sp. NBH77 contains a BGC with 100% homology to the antimycin/candicidin cluster and is a likely producer of these compounds. This may explain the broad-spectrum antimicrobial activity of this strain in the bioactivity assay (Benaud et al. 2021). Cytoxicity against tumour cell lines has not been examined here, but will form the basis of future work as genomic analyses of *Streptomyces* sp. NBSH44 found 100% homology to a BGC responsible for the production of the C-1027 enediyne subcluster from the C-1027 antitumour compound, suggesting production of a similar compound (van Lanen et al. 2008; Benaud et al. 2021).

With increasing emergence of pan-resistant infections, an urgency exists to find and develop new antimicrobials (Laxminarayan et al. 2013; Sun et al. 2018). The targeting of novel taxa and metabolically unique microbes from extreme environments remains a worthy approach for novel antimicrobial discovery (Harvey et al. 2015; Bérdy 2012; Pye et al. 2017). In culture-independent studies, PKS and NRPS sequence richness has been found to correspond to arid, nutrient poor soils, which contain an abundance of *Actinomycetota* (Charlop-Powers et al. 2014; Benaud et al. 2019), and functional NP diversity is influenced by geographical isolation, with endemic NP sequences observed in remote regions (Borsetto et al. 2019; Benaud et al. 2019). Antarctic desert soil microbiomes are underexplored and are rich in rare *Actinomycetota*, candidate phyla, and the NP-promising phyla *Planctomycetota*, *Chloroflexota* and *Verrucomicrobiota* (Crits-Christoph et al. 2018; Sharrar et al. 2019; Wiegand et al. 2020; Ji et al. 2017). Additionally, polar desert soil bacteria are metabolically adapted to life in an inhospitable and isolated environment (Obbels et al. 2016; Cary et al. 2010). In the isolation work conducted in this study, we showed that the HI soil indeed contains a diversity of culturable *Actinomycetota* with NP-synthesising capabilities, although the presence of NRPS and PKS genes and antibiotic activity appear to be somewhat less than typically seen in mesophilic *Streptomyces* (Ayuso-Sacido and Genilloud 2005; Kamat and Velho-Pereira 2011). This should be further investigated and may be due to a variety of factors including: Antarctic bacteria BGCs containing fewer of the NP domains amplified by the primer sets used here or experimental error, for example, while the genome for *Streptomyces* sp. NBH77 is known to harbour NRPS domains which should have been targeted by the primer set used in our PCR screening, they were undetected; different bioactivity profiles for NPs secreted by Antarctic soil biota; or suboptimal experimental conditions to stimulate the production of bioactive compounds. Obtaining this collection of pure isolates will now enable future extraction and chemical analysis of their secreted compounds.

## Conclusion

New antimicrobial compounds with novel modes of action are needed to combat widespread antibiotic resistance. Here, we isolated a collection of psychrotolerant antimicrobial-producing bacteria from the arid soils of Herring Island, East Antarctica, using two atypical culturing methods which exploit soil as a substrate for growth and use extended incubation times. We produced a library of 166 Antarctic strains, which included rarely cultured and potentially novel taxa. *Streptomyces* strains demonstrated antimicrobial activity, particularly against the Gram-positive test pathogens, *Bacillus* and *Staphylococcus*. Overall, we show that the *Actinomycetota*-rich soils of eastern Antarctica are worthy targets in the search for antibiotic-producing bacterial isolates with further work now required to characterise the natural product compounds synthesised by these bioactive strains.

## Supplementary Information

Below is the link to the electronic supplementary material.

Supplementary file1 (DOCX 55131 KB)

Supplementary file2 (DOCX 20 KB)

## Data Availability

Partial 16S rRNA gene sequences for the representative strains described in this study have been deposited to NCBI Genbank under the accession numbers MW050986—MW051016 and MW055683—MW055689. Complete genomic data for the following strains have been previously published (Benaud et al. 2021) and are available through NCBI under the accessions VUOJ00000000 (NBH48), CP045704-CP045705 (NBH77), CP043644-CP043649 (NBH84), CP043650-CP043651 (NBH87), CP043178 (NBSH8), CP045492-CP045495 (NBSH29) and CP045702-CP045703 (NBSH44). The Herring Island soil 16S rRNA gene amplicon sequencing datasets and associated environmental metadata are available through the Australian Antarctic Data (AAD) Centre, https://doi.org/10.4225/15/526F42ADA05B1.
