# Supplementary material for: Soil substrate culturing approaches recover diverse members of Actinomycetota from desert soils of Herring Island, East Antarctica
Source: Extremophiles. 2022 Jul 13;26(2):24. doi: 10.1007/s00792-022-01271-2 (PMC9279279; doi:10.1007/s00792-022-01271-2)
Supplement: Supplementary file 1 — Supplementary file1 (DOCX 55131 KB) [file 792_2022_1271_MOESM1_ESM.docx]

Supplementary Material for:

Soil-substrate culturing approaches recover diverse members of *Actinomycetota* from desert soils of Herring Island, East Antarctica

Nicole Benaud^1^, Devan S. Chelliah^1^, Sin Yin Wong^1^, Belinda C. Ferrari^1*^

^1^School of Biotechnology and Biomolecular Sciences, UNSW Sydney 2052, Australia

***Correspondence:** Associate Professor Belinda Ferrari [b.ferrari@unsw.edu.au](mailto:b.ferrari@unsw.edu.au)

# Supplementary Figures and Tables

## Supplementary Figures


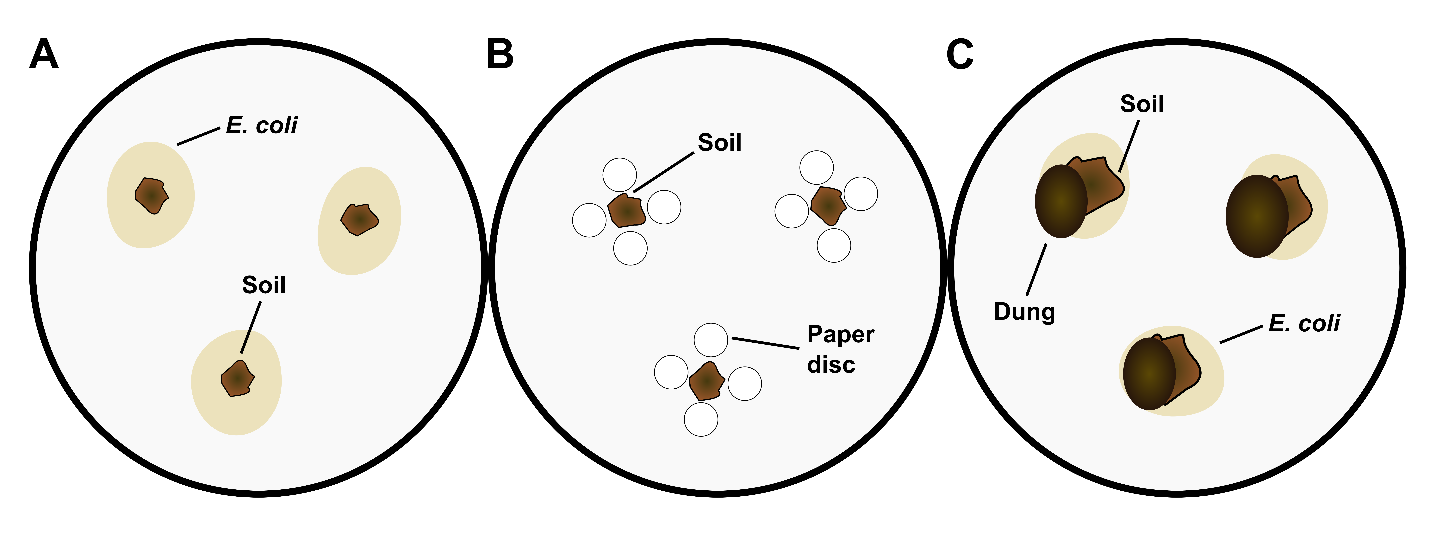


SI Figure 1. Direct soil culturing using *E. coli* lawn (A), and cellulose (B) baiting methods on WCX agar plates. Similar plates were set up for both soil preparations: soil pre-treated with mild heat and sonication, or untreated soil.


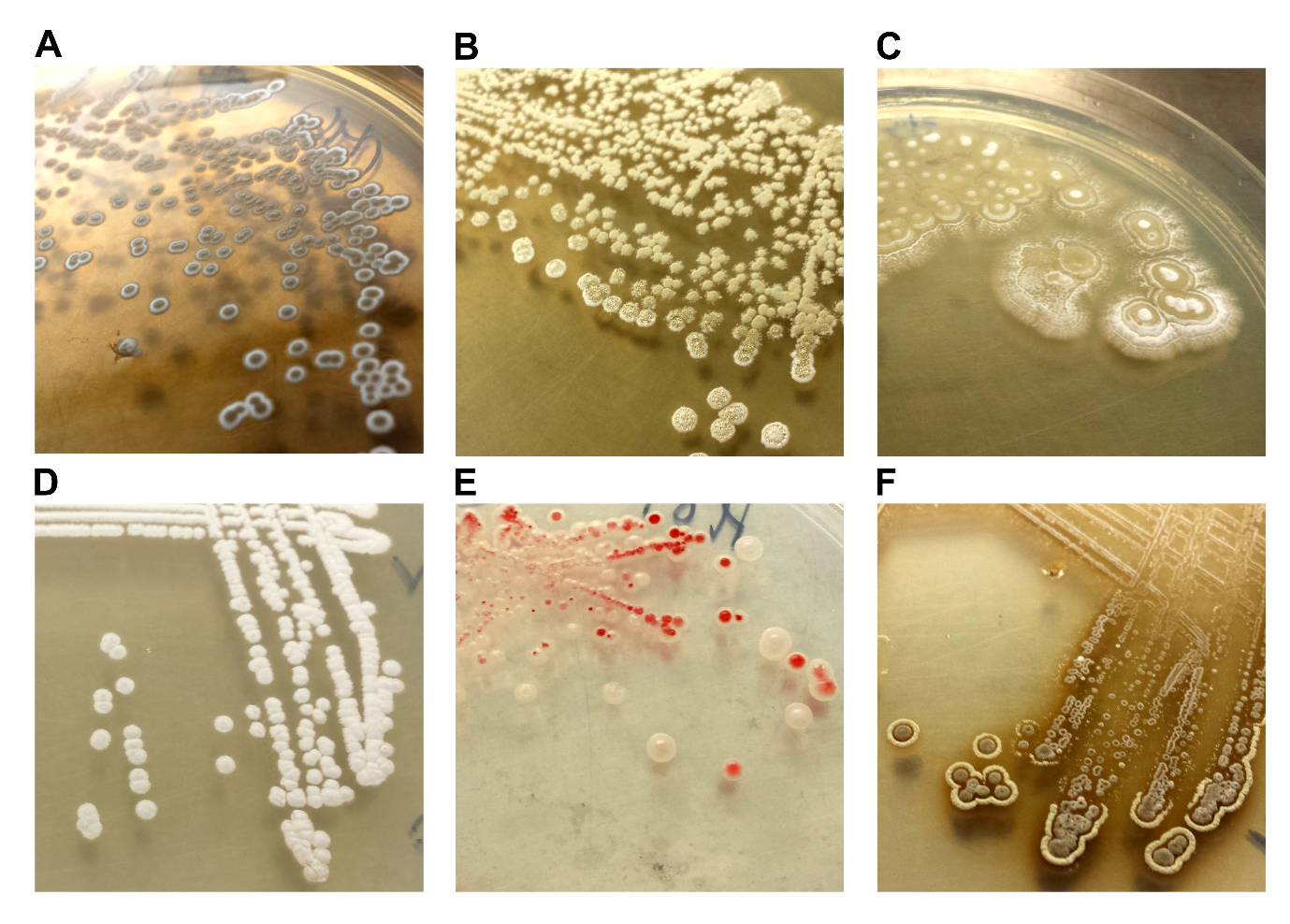


SI Figure 2. Colony morphology for six different *Streptomyces* strains. (A) *Streptomyces* sp. NBH20 formed white sporulation, with a dark melanin-like pigmentation which diffused into surrounding agar. (B) *Streptomyces* sp. NBH21 produced olive green/white sporulation with a tan-coloured diffused pigment. (C) For *Streptomyces* sp. NBH70, sporulation was white and diffused pigments were absent. (D) *Streptomyces* strains such as NBH41 were tan colonies with white to olive green sporulation and tan-coloured diffused melanin (E) *Streptomyces* sp. NBH81 produced red colony pigmentation and no diffused pigments. (F) *Streptomyces* sp. NBH77 formed ringed brown/tan sporulating colonies with a dark melanin-like pigment.


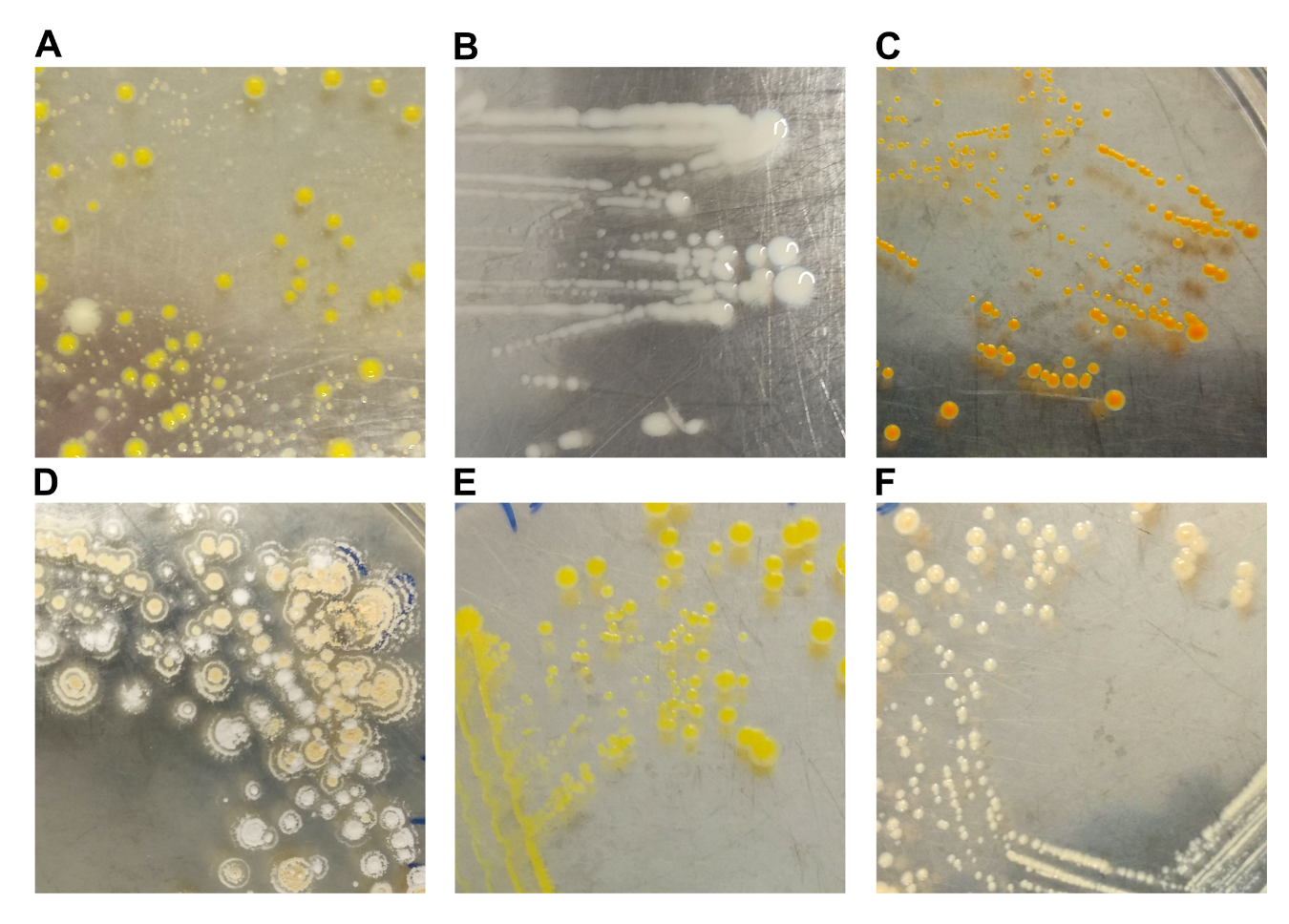


SI Figure 3. Bacteria cultured from HI by the cold-incubated SSMS. (A) The RAVAN media spread-plated communities were dominated by three main morphotypes; large yellow, large white, and smaller yellow-orange colonies. (B) Large white colonies were *Pseudarthrobacter* sp. (e.g. NBSH8). (C) Small yellow-orange colonies were *Rhodococcus* spp. (e.g. NBSH90). (D) Several *Streptomyces* spp. were recovered (e.g. NBSH44). (E) Large yellow colonies were *Arthrobacter* sp. (e.g. NBSH28). (F) *Mesorhizobium* sp. NBSH29 exhibited 98% similarity to known species.


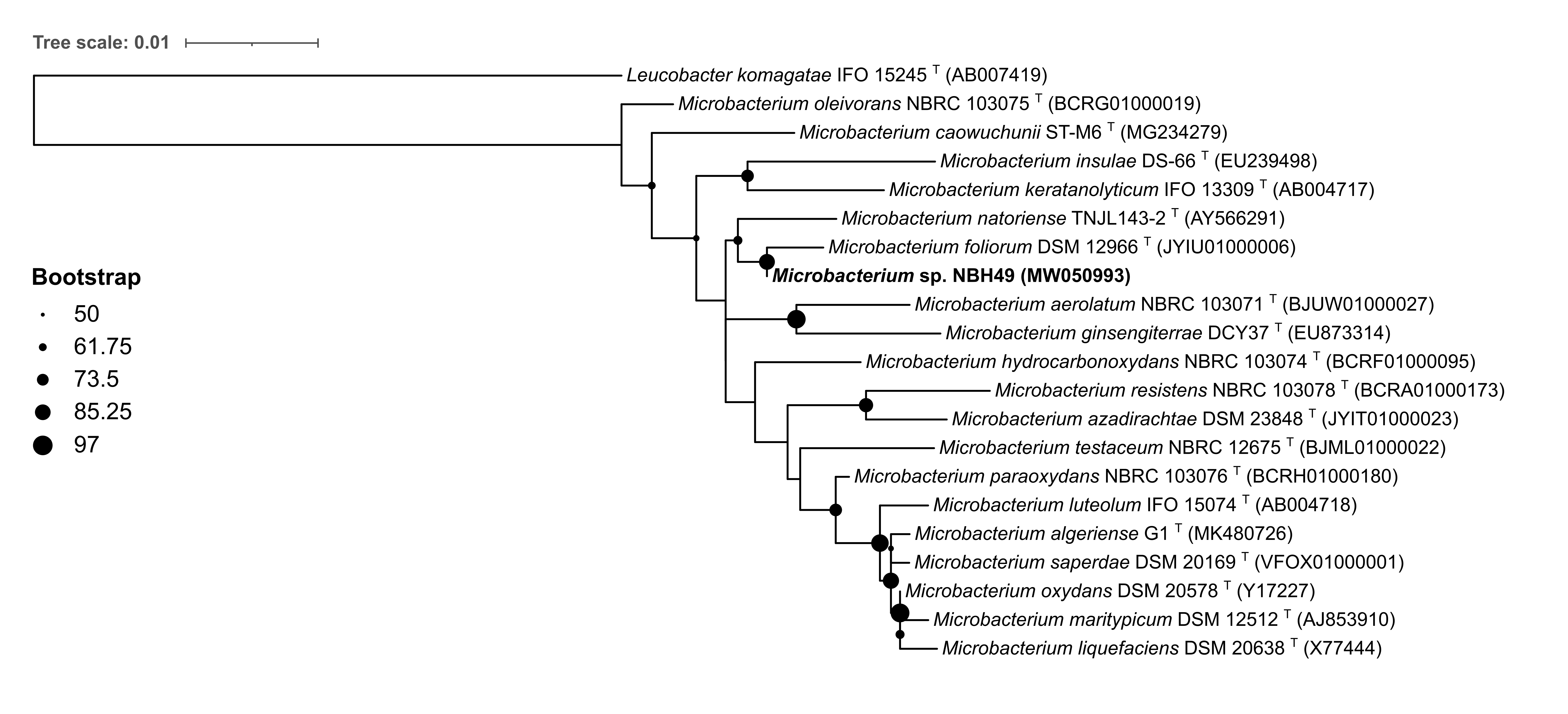


**SI Figure 4.** **Maximum-likelihood based on 16S rRNA gene sequences showing relationships between isolate NBH49 and the type strains of closely related *Microbacterium* species.** Bootstrap values greater than 50% are indicated. *Leucobacter komagatae* strain IFO 15245^T^ was used as an outgroup. The scale bar represented 0.01 substitutions per nucleotide position.


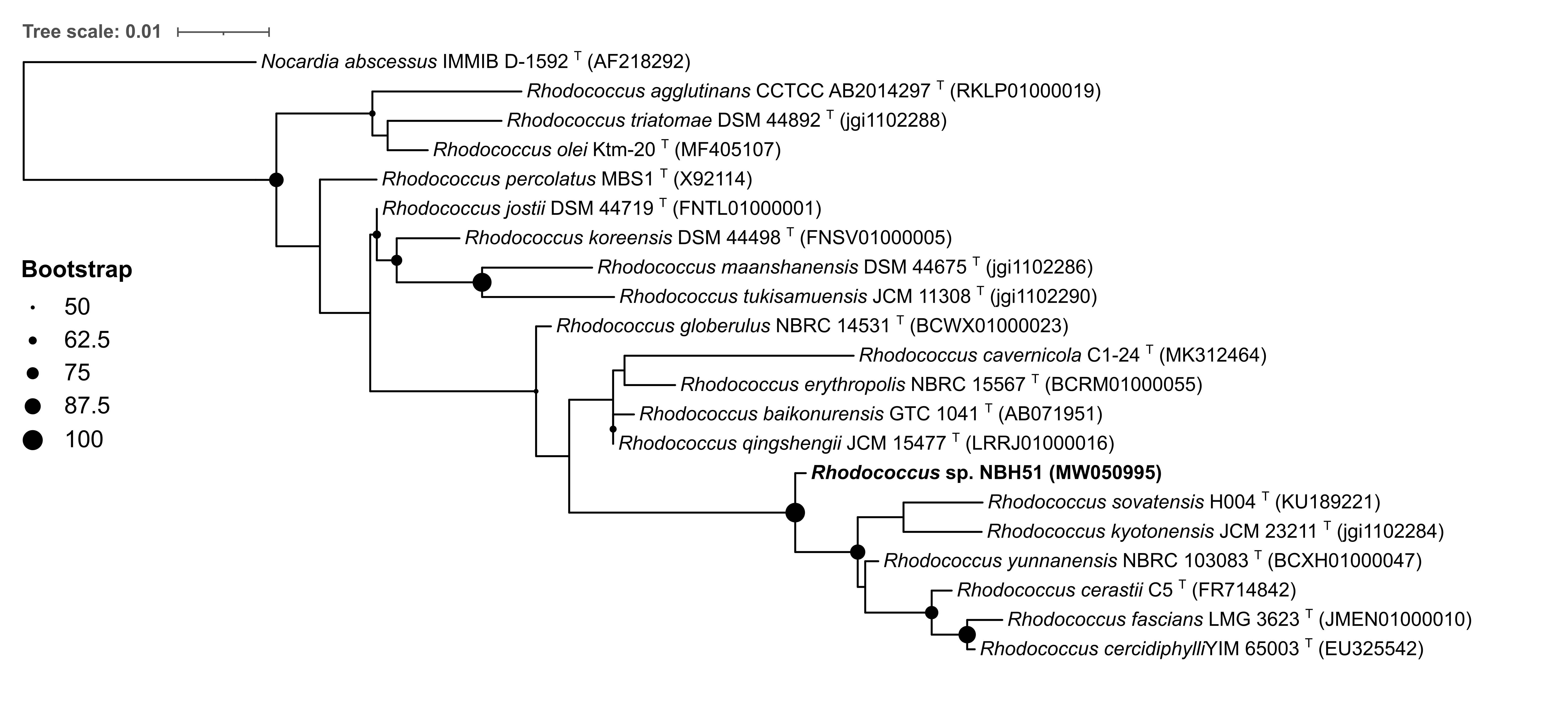


**SI Figure 5. Maximum-likelihood based on 16S rRNA gene sequences showing relationships between isolate NBH51 and the type strains of closely related *Rhodococcus* species.** Bootstrap values greater than 50% are indicated. *Nocardia abscessus* IMMIB D-1592^T^ was used as an outgroup. The scale bar represented 0.01 substitutions per nucleotide position.


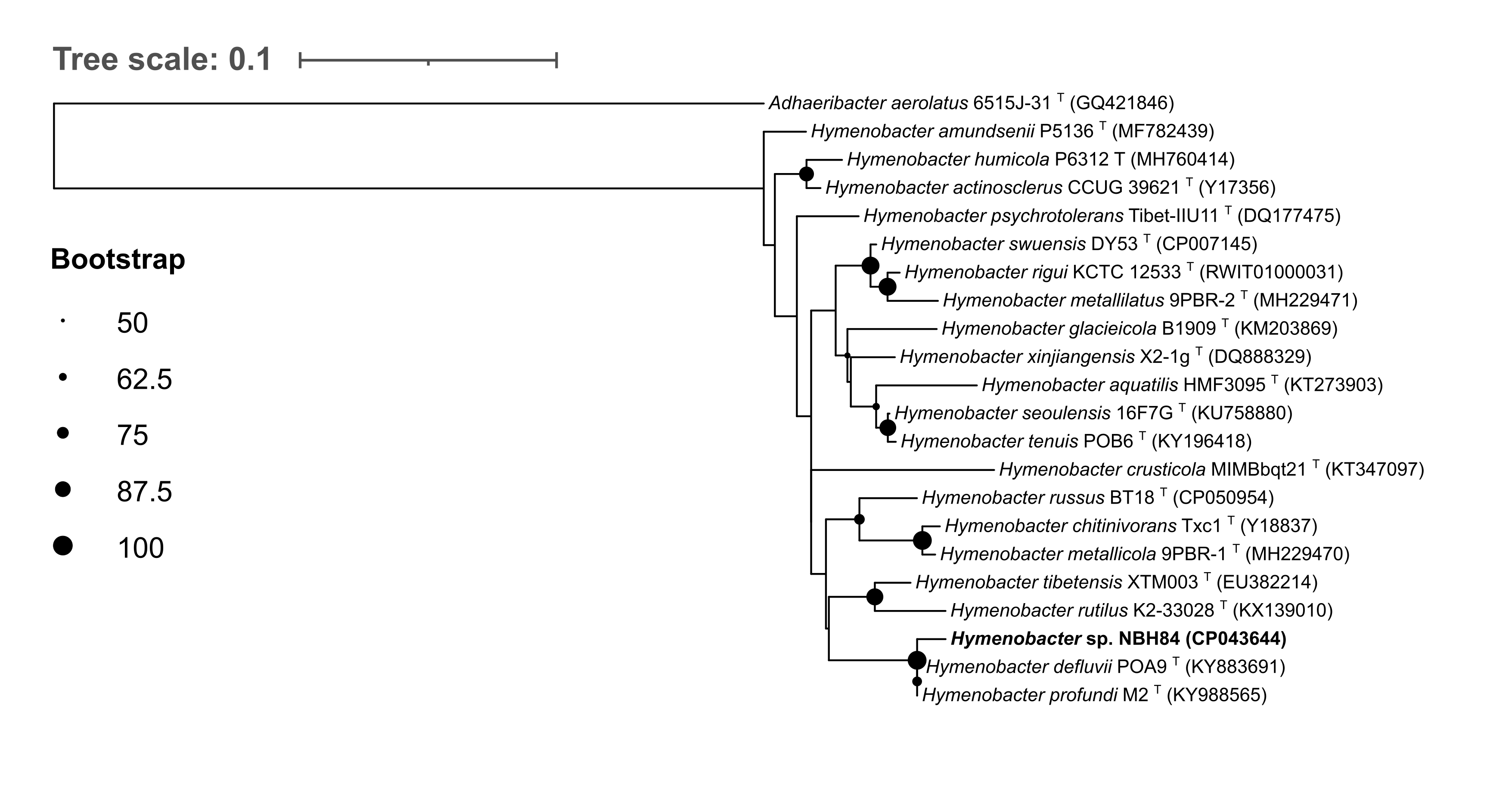


**SI Figure 6.** **Maximum-likelihood based on 16S rRNA gene sequences showing relationships between isolate NBH84 and the type strains of closely related *Hymenobacter* species.** Bootstrap values greater than 50% are indicated. *Adhaeribacter aerolatus* 6515J-31^T^ was used as an outgroup. The scale bar represented 0.1 substitutions per nucleotide position.


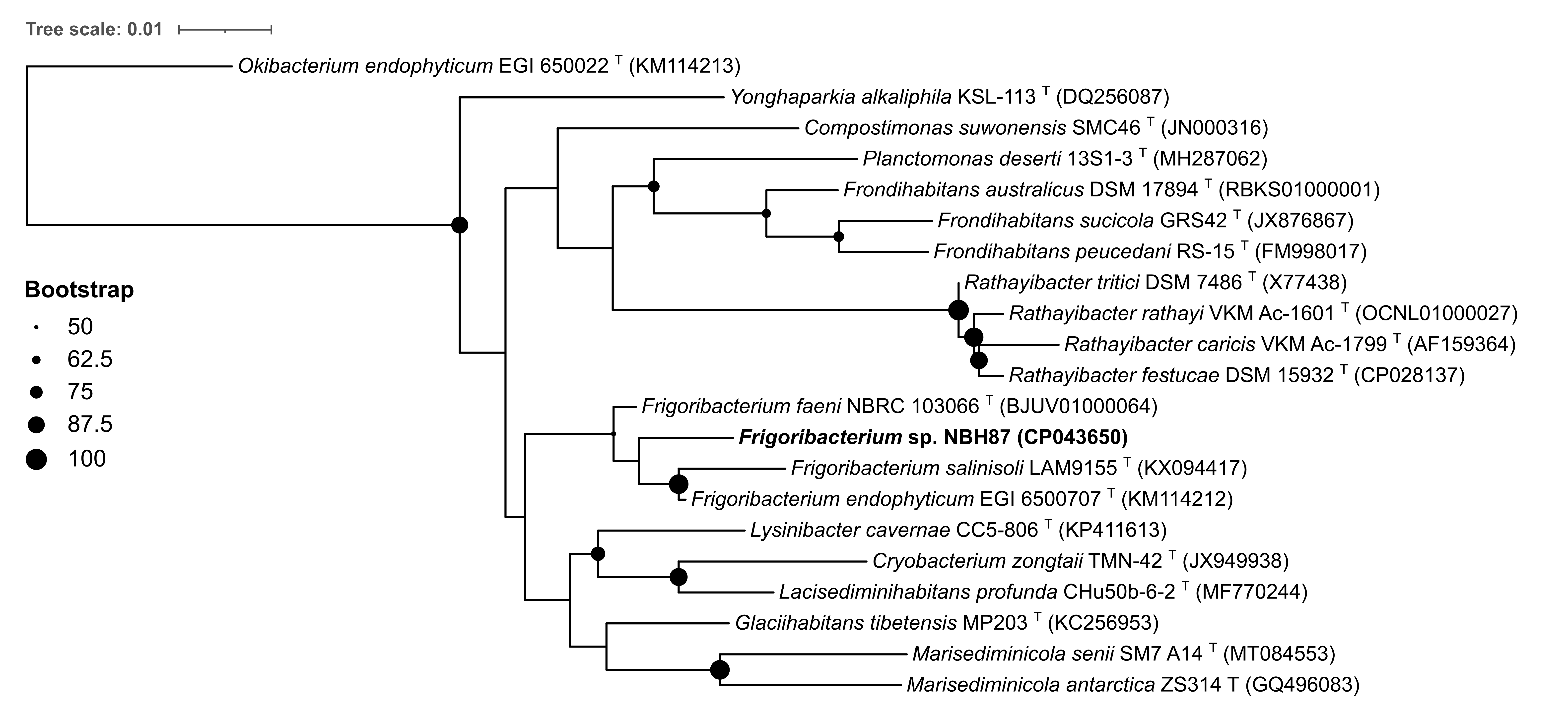


**SI Figure 7. Maximum-likelihood based on full 16S rRNA gene sequences showing relationships between isolate NBH87 and the type strains of closely related *Frigoribacterium* and other *Actinomycetota* genera such as *Rathayibacter*, *Lysinibacter* and *Glacihabitans*.** Bootstrap values greater than 50% are indicated. *Okibacterium endophyticum* strain EGI 650022^T^ was used as an outgroup. The scale bar represented 0.01 substitutions per nucleotide position.


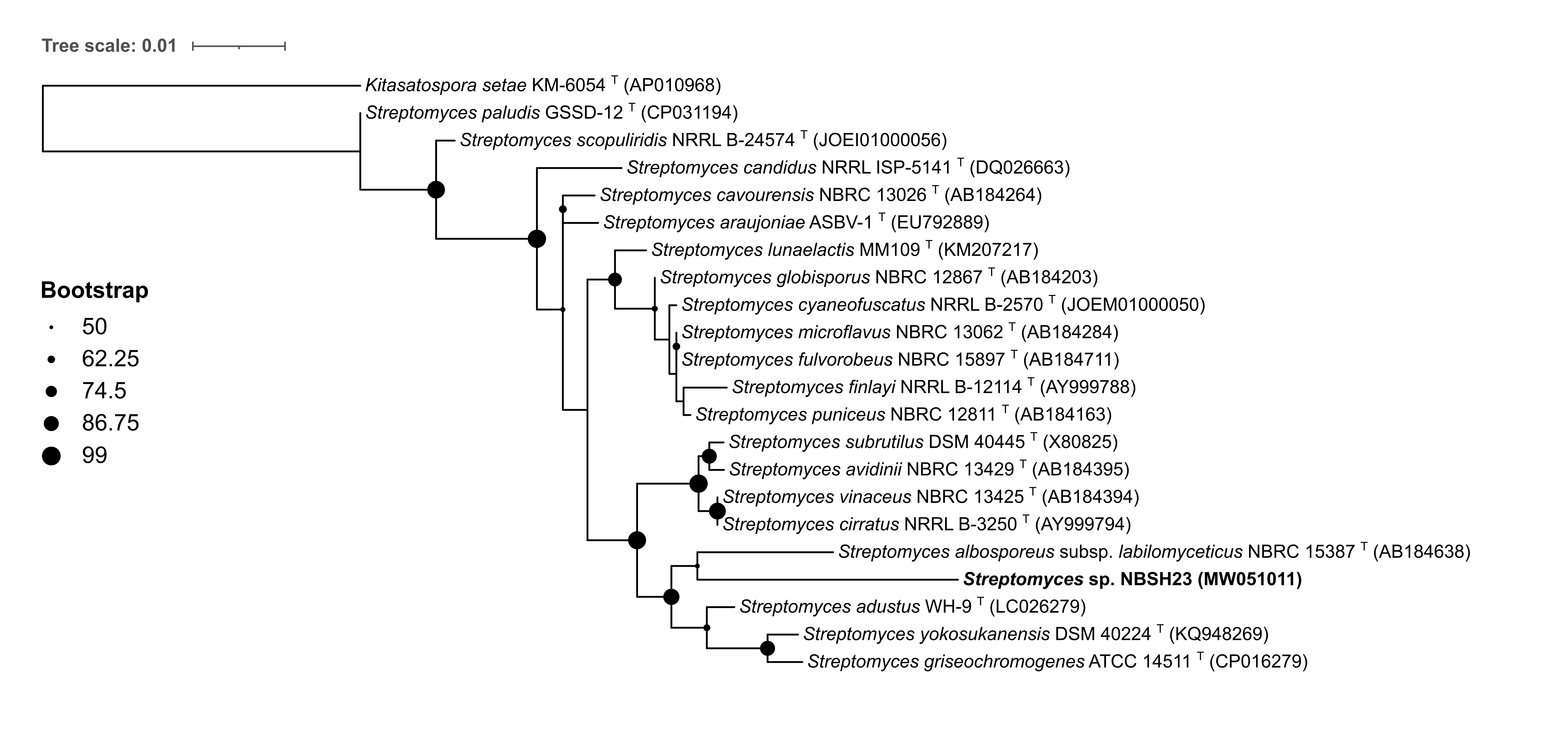


**SI Figure 8.** **Maximum-likelihood based on 16S rRNA gene sequences showing relationships between isolate NBSH23 and the type strains of closely related *Streptomyces* species.** Bootstrap values greater than 50% are indicated. *Kitasatospora setae* strain KM-6054^T^ was used as an outgroup. The scale bar represented 0.01 substitutions per nucleotide position.


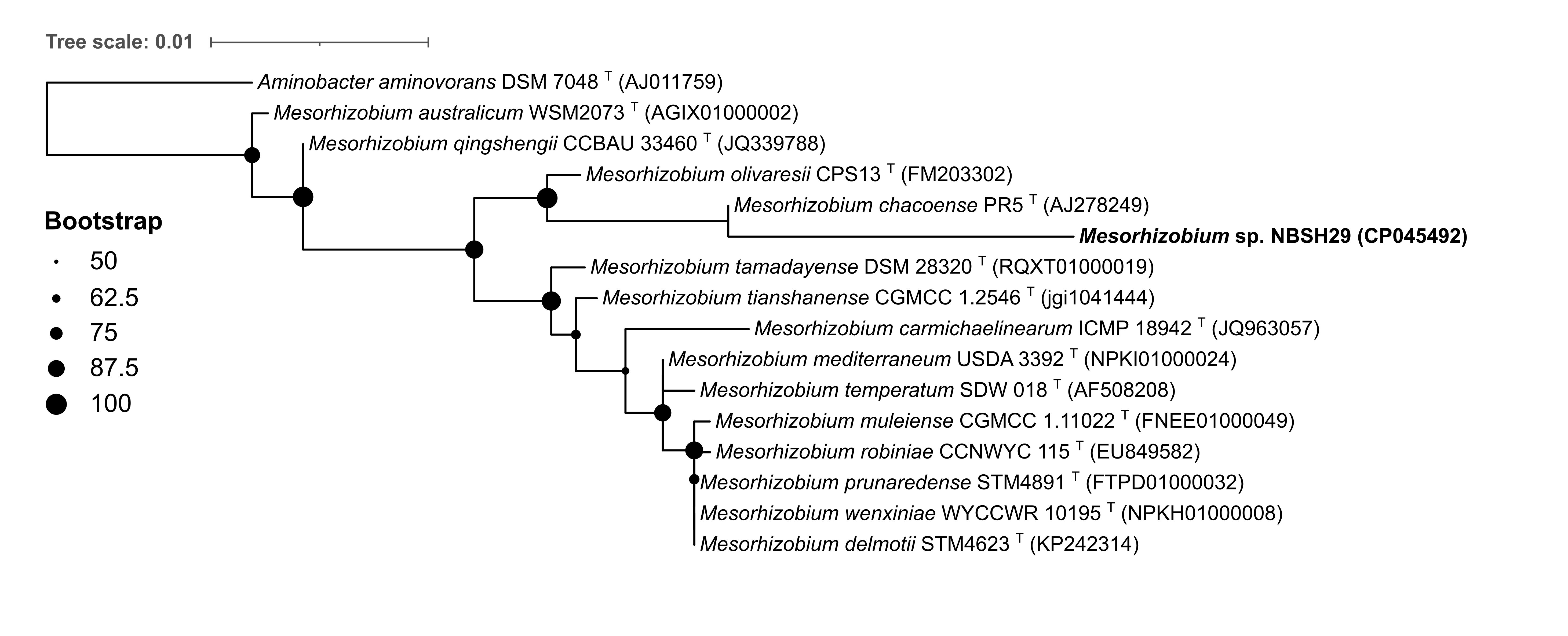


**SI Figure 9.** **Maximum-likelihood based on 16S rRNA gene sequences showing relationships between isolate NBSH29 and the type strains of closely related *Mesorhizobium* species.** Bootstrap values greater than 50% are indicated. *Aminobacter aminovorans* DSM 7048^T^ was used as an outgroup. The scale bar represented 0.01 substitutions per nucleotide position.


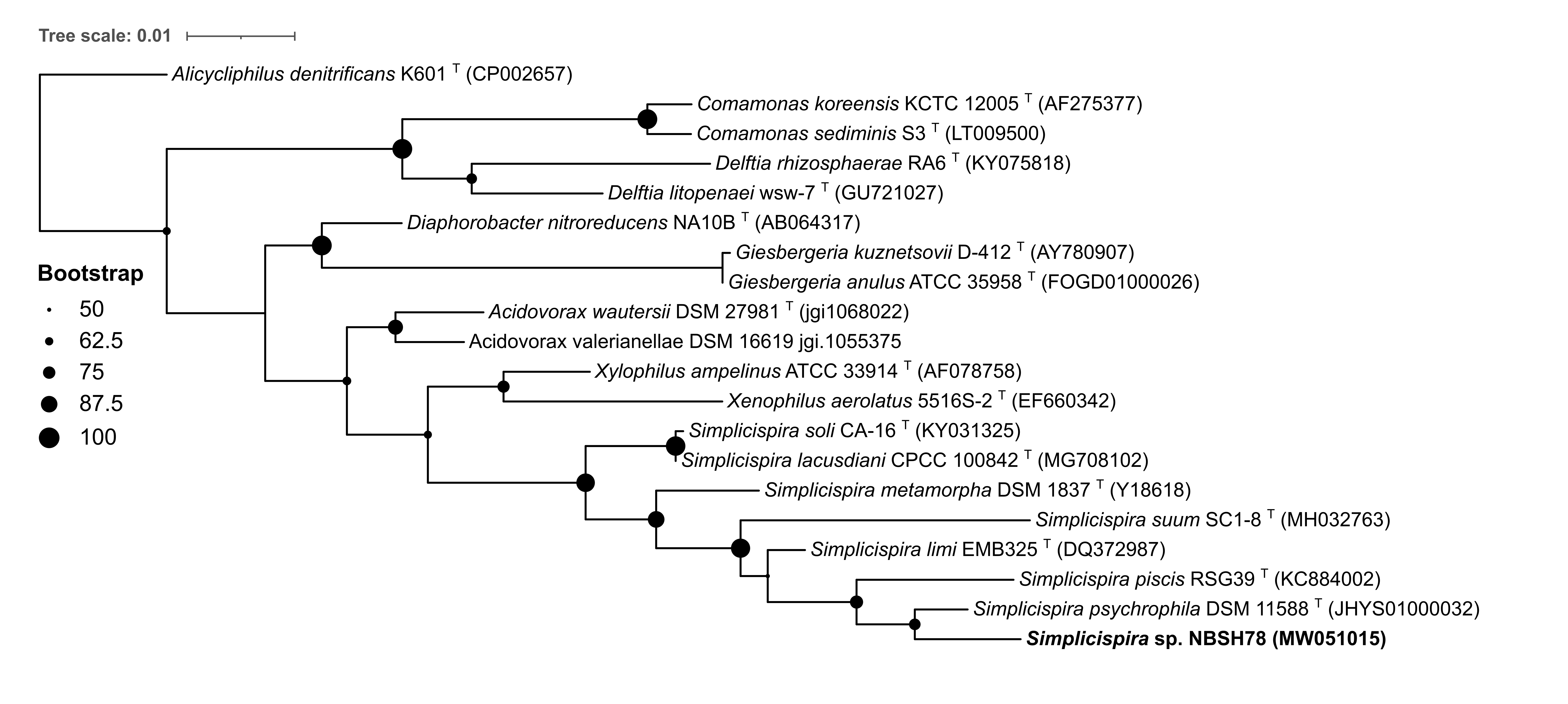


**SI Figure 10. Maximum-likelihood based on 16S rRNA gene sequences showing relationships between isolate NBSH78 and the type strains of closely related *Simplicispira* species and *Gammaproteobacteria* genera such as *Xylophilus* and *Acidivorax*.** Bootstrap values greater than 50% are indicated. *Alicycliphilus denitrificans* K601^T^ was used as an outgroup. The scale bar represented 0.01 substitutions per nucleotide position.

**
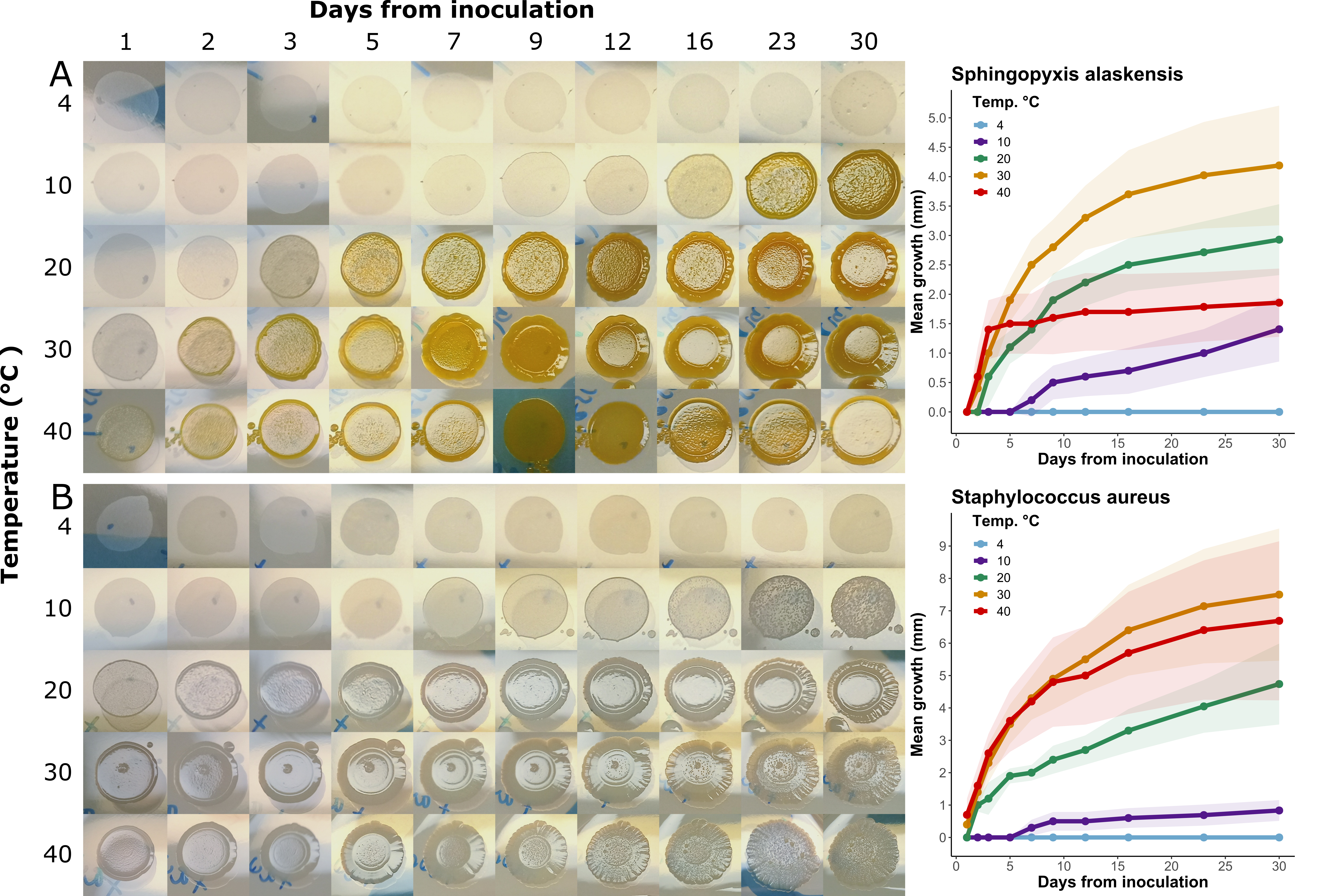
**

**SI Figure 11. Growth profiles for control strains at temperatures 4, 10, 20, 30 and 40°C over a 30 day incubation period.** For each time point, charts show mean of 21 replicates at each incubation temperature. **(A)** *Sphingopyxis alaskensis* RB2256 is a cold-adapted strain isolated from Alaskan sea water and capable of growth across a wide temperature range (5 - 45°C) (Eguchi et al. 1996). **(B)** *Staphylococcus aureus* ATCC 25923, an opportunistic human pathogen with optimal growth of 30 - 37℃ (Cowan et al. 1954).

**
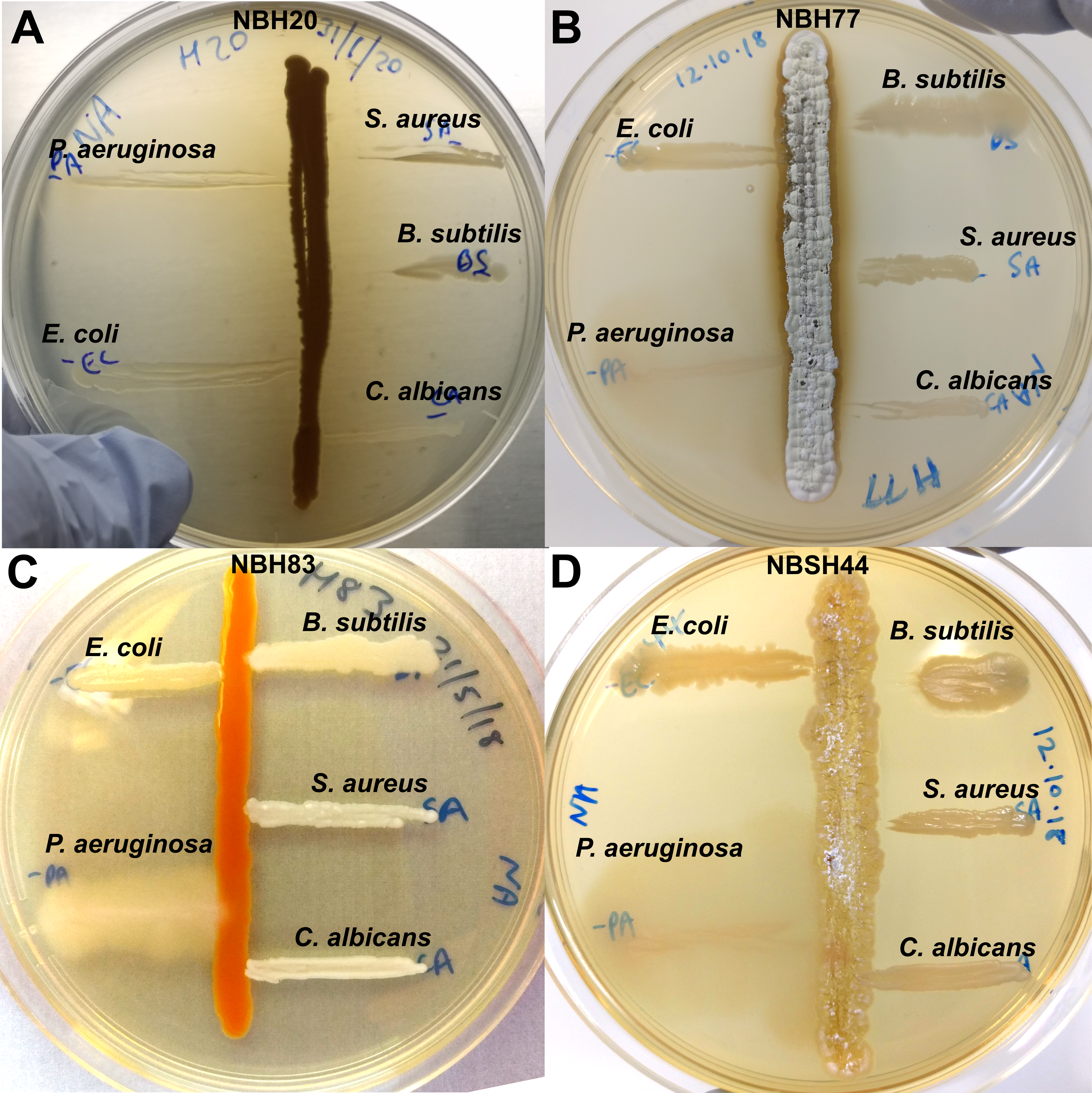
**

SI Figure 12. Cross-streak antimicrobial assay screening for bacterial isolates. (A) *Streptomyces* sp. NBH20 displayed measurable activity against Gram-positive pathogens *Bacillus subtilis* and *Staphylococcus aureus*, and inhibited growth for Gram-negative pathogens *Pseudomonas aeruginosa* and *Escherichia coli*, as well as the yeast, *Candida albicans*. (B) *Streptomyces* sp. NBH77 was active against Gram-positive pathogens and yeast. (C) *Sphingomonas* sp. NBH83 displayed no inhibition of test pathogens. (D) Cold-incubated, SSMS-grown isolate *Streptomyces* sp. NBSH44 showed measurable activity against Gram-positive pathogens, and inhibition of *E. coli.*

## Supplementary Tables

**SI Table 1.** **Location and soil characteristics for selected Antarctic soils.**

|  | **Herring Island soil** | **References** |
| --- | --- | --- |
| **AAD Barcode** | 36815 | van Dorst et al. 2014; Siciliano et al. 2014; https://doi.org/10.4225/15/526F42ADA05B1 |
| **Transect/Distance** | T2/ 200m |  |
| **Antarctic Region** | Windmill Is. |  |
| **Moisture (%)** | 3.2 |  |
| **Total Carbon (ppm)** | 600 |  |
| **Total Nitrogen (ppm)** | 130 |  |
| **pH** | 6.6 |  |
| **Latitude/Longitude** | 66° 24' 41”S, 110° 39' 30”E |  |
| **Geological  composition †** | Garnet-bearing granite gneiss | Paul et al. 1995;  Bailey et al. 2016 |

AAD: Australian Antarctic Division

**SI Table 2 (*Description*).**

Sheet 1: OTU table and assigned taxonomy for Herring Island soils

Sheet 2: Differential abundance analysis using ANCOM-BC for the 0 m distance samples

Sheet 3: Differential abundance analysis using ANCOM-BC for the 2 m distance samples

Sheet 4: Differential abundance analysis using ANCOM-BC for the 100 m distance samples

Sheet 5: Differential abundance analysis using ANCOM-BC for the 102 m distance samples

Sheet 5: Differential abundance analysis using ANCOM-BC for the 200 m distance samples

Sheet 5: Differential abundance analysis using ANCOM-BC for the 202 m distance samples

**SI Table 3 AutoMLST analysis data (*Description*)**

Sheet 1: NBH84 genome comparisons

Sheet 2: NBH84 Core genes

Sheet 3: NBH87 genome comparisons

Sheet 4: NBH87 Core genes

Sheet 5: NBSH29 genome comparisons

Sheet 6: NBSH29 core genes

## Supplementary References

Bailey, B.T., Morgan, P.J., and Lackie, M.A. (2016). An assessment of the gravity signature of the Windmill Islands, East Antarctica. *Antarctic Science* 28(2)**,** 115-126. doi: 10.1017/S0954102015000565.

Paul, E., Stüwe, K., Teasdale, J., and Worley, B. (1995). Structural and metamorphic geology of the Windmill Islands, east Antarctica: Field evidence for repeated tectonothermal activity. *Australian Journal of Earth Sciences* 42(5)**,** 453-469. doi: 10.1080/08120099508728216.

Siciliano, S.D., Palmer, A.S., Winsley, T., Lamb, E., Bissett, A., Brown, M.V., et al. (2014). Soil fertility is associated with fungal and bacterial richness, whereas pH is associated with community composition in polar soil microbial communities. *Soil Biology and Biochemistry* 78**,** 10-20. doi: 10.1016/j.soilbio.2014.07.005.

van Dorst, J., Bissett, A., Palmer, A.S., Brown, M., Snape, I., Stark, J.S., et al. (2014). Community fingerprinting in a sequencing world. *FEMS Microbiology Ecology* 89(2)**,** 316-330. doi: 10.1111/1574-6941.12308.
