# Supplementary material for: Soil substrate culturing approaches recover diverse members of Actinomycetota from desert soils of Herring Island, East Antarctica
Source: Extremophiles. 2022 Jul 13;26(2):24. doi: 10.1007/s00792-022-01271-2 (PMC9279279; doi:10.1007/s00792-022-01271-2)
Supplement: Supplementary file 2 — Supplementary file2 (DOCX 20 KB) [file 792_2022_1271_MOESM2_ESM.docx]

Example page **Supplementary Table 2**

| Count | #OTU ID | Kingdom | Phylum | Class | Order | Family | Genus | HI/T1/0 | HI/T2/0 | HI/T3/0 | HI/T1/2 | HI/T2/2 | HI/T3/2 | HI/T1/100 | HI/T2/100 |
| --- | --- | --- | --- | --- | --- | --- | --- | --- | --- | --- | --- | --- | --- | --- | --- |
| 21008 | AMD_16S_OTUa_12 | Bacteria(100) | Actinobacteriota(100) | Actinobacteria(100) | Pseudonocardiales(100) | Pseudonocardiaceae(100) | Crossiella(100) | 2222 | 1658 | 963 | 875 | 1447 | 473 | 2296 | 1868 |
| 6726 | AMD_16S_OTUa_23 | Bacteria(100) | Acidobacteriota(100) | Blastocatellia(100) | Blastocatellales(100) | Blastocatellaceae(100) | Blastocatella(100) | 365 | 221 | 464 | 427 | 532 | 384 | 242 | 286 |
| 4695 | AMD_16S_OTUa_60 | Bacteria(100) | Actinobacteriota(100) | Rubrobacteria(100) | Rubrobacterales(100) | Rubrobacteriaceae(100) | Rubrobacter(100) | 296 | 395 | 308 | 340 | 516 | 234 | 276 | 515 |
| 4640 | AMD_16S_OTUa_8 | Bacteria(100) | Proteobacteria(100) | Gammaproteobacteria(100) | Pseudomonadales(100) | Moraxellaceae(100) | Psychrobacter(100) | 2 | 22 | 42 | 0 | 291 | 197 | 3 | 28 |
| 3236 | AMD_16S_OTUa_38 | Bacteria(100) | Acidobacteriota(100) | Blastocatellia(100) | Blastocatellales(100) | Blastocatellaceae(100) | Blastocatellaceae_unclassified(100) | 114 | 137 | 173 | 141 | 206 | 258 | 196 | 173 |
| 3110 | AMD_16S_OTUa_251 | Bacteria(100) | Chloroflexi(100) | KD4-96(100) | KD4-96_or(100) | KD4-96_fa(100) | KD4-96_ge(100) | 163 | 264 | 254 | 318 | 91 | 240 | 143 | 283 |
| 3047 | AMD_16S_OTUb_1635 | Bacteria(100) | Acidobacteriota(100) | Blastocatellia(100) | Blastocatellales(100) | Blastocatellaceae(100) | Blastocatella(100) | 58 | 45 | 149 | 195 | 100 | 205 | 51 | 85 |
| 2983 | AMD_16S_OTUa_42 | Bacteria(100) | Actinobacteriota(100) | Actinobacteria(100) | Actinobacteria_unclassified(100) | Actinobacteria_unclassified(100) | Actinobacteria_unclassified(100) | 567 | 523 | 66 | 146 | 345 | 18 | 100 | 280 |
| 2244 | AMD_16S_OTUa_80 | Bacteria(100) | Actinobacteriota(100) | Actinobacteria(100) | Propionibacteriales(100) | Nocardioidaceae(100) | Nocardioidaceae_unclassified(100) | 21 | 3 | 39 | 43 | 1 | 88 | 9 | 0 |

Example page **Supplementary Table 3**

| **#Query_org** | **Reference_assembly** | **Ref_name** | **MASH_distance** | **Estimated_ANI** | **P-value** | **Genus** | **Order** | **Type_strain** | **Included in tree** |
| --- | --- | --- | --- | --- | --- | --- | --- | --- | --- |
| QS--Hymenobacter_NBH84 | GCF_900142395 | Hymenobacter_psychrotolerans_DSM_18569 | 0.1559 | 0.8441 | 0 | Hymenobacter | Cytophagales | TRUE | Y |
| QS--Hymenobacter_NBH84 | GCF_000576555 | Hymenobacter_swuensis_DY53 | 0.2078 | 0.7922 | 0 | Hymenobacter | Cytophagales | TRUE | Y |
| QS--Hymenobacter_NBH84 | GCF_900187375 | Hymenobacter_gelipurpurascens | 0.2101 | 0.7899 | 0 | Hymenobacter | Cytophagales | TRUE | Y |
| QS--Hymenobacter_NBH84 | GCF_000382225 | Hymenobacter_aerophilus_DSM_13606 | 0.2117 | 0.7883 | 0 | Hymenobacter | Cytophagales | TRUE | Y |
| QS--Hymenobacter_NBH84 | GCF_900107135 | Hymenobacter_psychrophilus | 0.2117 | 0.7883 | 0 | Hymenobacter | Cytophagales | TRUE | Y |
| QS--Hymenobacter_NBH84 | GCF_900141805 | Hymenobacter_daecheongensis_DSM_21074 | 0.2117 | 0.7883 | 0 | Hymenobacter | Cytophagales | TRUE | Y |
| QS--Hymenobacter_NBH84 | GCF_001816125 | Hymenobacter_coccineus | 0.2168 | 0.7832 | 0 | Hymenobacter | Cytophagales | TRUE | Y |
| QS--Hymenobacter_NBH84 | GCF_001816165 | Hymenobacter_glacialis | 0.2186 | 0.7814 | 0 | Hymenobacter | Cytophagales | TRUE | Y |
| QS--Hymenobacter_NBH84 | GCF_900188255 | Hymenobacter_mucosus | 0.2205 | 0.7795 | 0 | Hymenobacter | Cytophagales | TRUE | Y |
| QS--Hymenobacter_NBH84 | GCF_900176135 | Hymenobacter_roseosalivarius_DSM_11622 | 0.2245 | 0.7755 | 0 | Hymenobacter | Cytophagales | TRUE | Y |
| QS--Hymenobacter_NBH84 | GCF_000420705 | Hymenobacter_norwichensis_DSM_15439 | 0.2267 | 0.7733 | 0 | Hymenobacter | Cytophagales | TRUE | Y |
| QS--Hymenobacter_NBH84 | GCF_001816145 | Hymenobacter_lapidarius | 0.2267 | 0.7733 | 0 | Hymenobacter | Cytophagales | TRUE | Y |
| QS--Hymenobacter_NBH84 | GCF_001507645 | Hymenobacter_sedentarius | 0.2338 | 0.7662 | 0 | Hymenobacter | Cytophagales | TRUE | Y |
| QS--Hymenobacter_NBH84 | GCF_000972495 | Hymenobacter_terrenus | 0.2506 | 0.7494 | 0 | Hymenobacter | Cytophagales | TRUE | Y |
| QS--Hymenobacter_NBH84 | GCF_000373265 | Pontibacter_roseus_DSM_17521 | 0.3066 | 0.6934 | 0 | Pontibacter | Cytophagales | TRUE | Y |
| QS--Hymenobacter_NBH84 | GCF_001647275 | Rufibacter_ruber | 0.3066 | 0.6934 | 0 | Rufibacter | Cytophagales | TRUE | Y |
| QS--Hymenobacter_NBH84 | GCF_900188175 | Pontibacter_ummariensis | 0.329 | 0.671 | 0 | Pontibacter | Cytophagales | TRUE | Y |
| QS--Hymenobacter_NBH84 | GCF_001310085 | Rufibacter_tibetensis | 0.3396 | 0.6604 | 0 | Rufibacter | Cytophagales | TRUE | Y |
| QS--Hymenobacter_NBH84 | GCF_900129015 | OG--Cnuella_takakiae | 0.3533 | 0.6467 | 0 | Cnuella | Chitinophagales | TRUE | Y |
